# Fibronectin's tensional state as a mechanical signature of fibrotic extracellular matrix in idiopathic pulmonary fibrosis models

**DOI:** 10.1016/j.mbplus.2026.100204

**Published:** 2026-08-25

**Authors:** Konstantin M.P. Wolf, Emmanouil Angelidakis, Daria Künzli, David Lauer, Matthias Brunner, Alessia Cambria, Divya Vats, Britta Maurer, Viola Vogel, Mamta Chabria

**Affiliations:** a Laboratory of Applied Mechanobiology, Institute of Translational Medicine, Department of Health Sciences and Technology, ETH Zurich, Switzerland; b Department of Rheumatology and Immunology, Inselspital, Bern University Hospital, University of Bern, Bern, Switzerland; c Lung Precision Medicine (LPM), Department of BioMedical Research (DBMR), University of Bern, Bern, Switzerland

**Keywords:** Extracellular matrix, Fibronectin, Mechanics, Idiopathic pulmonary fibrosis

## Abstract

Idiopathic pulmonary fibrosis (IPF) is a progressive and fatal disease with limited treatment options. Emerging evidence suggests that the composition and mechanics of the extracellular matrix (ECM) play a crucial role in IPF pathogenesis; however, biomarkers that indicate altered ECM signatures are scarce. To demonstrate a direct relationship between ECM fiber tension and fibrosis progression, we established an *in vitro* fibrosis assay using patient-derived fibroblasts and the tension-sensitive fibronectin-binding peptide FnBPA5, whose multivalent binding to fibronectin is impaired by fiber strain. The system was validated using known fibrosis biomarkers at the protein and mRNA levels. Loss of fibronectin fiber tension not only acts as a marker for fibrosis, but as shown here, tension can be restored upon treatment with antifibrotic drugs, concomitant with improved biomarker data. The loss of fibronectin fiber tension was further corroborated *ex vivo* by probing fibrotic lung cryosections from bleomycin-treated mice with FnBPA5. Taken together, our results highlight the mechanical state of fibronectin fibers as a promising biomarker for IPF.

## Highlights

• IPF is a progressive and fatal disease, with the extracellular matrix (ECM) playing a key role in its pathology.
• Limited biomarkers to map changes in ECM architecture as the disease progresses.
• Fibronectin fibers progressively relax as fibrosis is established *in vivo*.
• *In vitro* fibrosis assay recapitulates fibronectin fiber relaxation upon fibrosis onset.
• Fibronectin fiber tension can be restored upon antifibrotic drug treatment.

## Introduction

Interstitial lung diseases (ILDs) encompass a broad spectrum of pulmonary disorders characterized by chronic inflammation and excessive scarring of lung tissue [1]. While the clinical presentation of ILDs is heterogeneous, patients with a progressive fibrotic phenotype (PF-ILD) experience significantly worse outcomes, with a median survival of only 3–5 years [2]. Idiopathic pulmonary fibrosis (IPF) is the most prevalent form of PF-ILD [3], sharing clinical and mechanistic features with other fibrosing ILDs. IPF affects approximately 0.9 to 9 individuals per 100,000 in Europe and North America [4], [5], and its global incidence is rising, likely driven by demographic aging [6], [7]. IPF is an irreversible condition [6], [8], and treatment options remain limited. Despite advances in understanding IPF pathogenesis and associated risk factors, a major translational gap has resulted in multiple failures in late-stage clinical trials [9], [10]. There is an urgent need to bridge this gap with physiologically relevant *in vitro* models for robust preclinical discovery that recapitulate the various hallmarks of fibrotic progression, including changes in the extracellular matrix (ECM).

Like most progressive fibrotic disorders, IPF is driven by pathological remodeling at the tissue level, mostly through excessive deposition of ECM, which impairs alveolar architecture and function [11]. While the exact mechanism of disease onset remains unknown, initial injury to the alveolar epithelium is believed to trigger an inflammatory response involving immune cell infiltration, which drives dysregulated tissue repair of the lung parenchyma over an extended time [12], [13], [14], [15]. The sustained inflammatory environment triggers excessive differentiation of fibroblasts into myofibroblasts (fibroblast-to-myofibroblast transition, FMT), which overproduce ECM components that progressively make the surrounding ECM denser [16]. Increased ECM stiffness further sustains profibrotic signaling through activation of the transforming growth factor beta 1 (TGF-β1) cytokine, by release from ECM reservoirs, creating a vicious feedback cycle [17], [18]. High levels of tissue inhibitors of metalloproteinases (TIMPs) produced by fibroblasts prevent the natural breakdown and clearance of scar tissue [19], further aggravating this progressive disease.

The known ECM components that are key drivers of IPF pathology include collagens, hyaluronan, tenascin-C, and fibronectin (FN) [20], [21]. Fibrillar collagen is essential for tissue homeostasis as it is the most abundant type of ECM component in the lung and provides tensile strength and tissue functionality as the primary load-bearing element [22]. In IPF pathogenesis, collagen accumulates around myofibroblasts within fibroblastic foci, resulting in increased stiffness within these regions [23], [24], [25] with insufficiently understood consequences for the surrounding ECM. Cell culture experiments have demonstrated that the onset of collagen fibrillogenesis depends on nucleation by FN templating [26], [27]. FN fibers are of further interest as they interact with their environment in a multifaceted manner by providing a plethora of binding sites for many growth factors, cytokines, and other matrix molecules [28], [29]. The binding repertoire can be even further expanded by mechanical forces, as stretching of FN fibers can destroy (multivalent) epitopes or reveal previously cryptic binding sites [27], [29], [30], [31], [32].

Although research has started highlighting the prominent role of ECM aberrations as drivers of various diseases [33], [34], reliable tools and methods that can capture the mechanical state of ECM fibers are still lacking. Additionally, few *in vitro* models have successfully recapitulated the dynamic evolution of ECM architecture and mechanics from disease onset to advanced fibrotic stages. *In vivo*, the bleomycin-induced mouse model remains the most widely used standard for studying pulmonary fibrosis [35]. However, its translational relevance is constrained by poor scalability, limited humanized features and phenotypic heterogeneity, and its reliance on a defined etiology (*i.e.*, bleomycin treatment), which stands in contrast to the idiopathic nature of IPF [36]. These shortcomings underscore the urgent need for scalable, mechanistically informative *in vitro* models to bridge the translational gap in preclinical research.

The development of the mechano-sensitive FnBPA5 peptide probe has enabled the visualization of FN fiber strain at the molecular scale and at the tissue level, both *in vitro* and in *ex vivo* stains [37], [38], [39], [40], making it a key tool for mapping FN fiber tension in health and disease. The peptide (schematically depicted in Fig. S1, adapted from Arnoldini *et al*. [37]) binds FN's N-terminal type I domains (FnI_2–5_) with nanomolar affinity [37], [41], [42]. Mechanical strain disrupts this multivalent epitope, causing a structural mismatch that reduces binding [41], [42]. Steered molecular dynamics showed that tensile forces can elongate the FnI_1–5_ fragment by >30% via stretching linker residues [42], and fiber stretching assays confirmed preferential FnBPA5 binding to relaxed FN fibers with gradually decreasing affinity as fiber strain increased [37]. In cell culture, labeling with a fibronectin-FRET (Fluorescence resonance energy transfer) strain probe versus FnBPA5 showed strong correlation over a wide strain range, providing orthogonal validation [37], [43], which was normalized by counterstaining with a FN polyclonal antibody that binds to total FN irrespective of physical state [37], [41], [42], [44]. Probing with FnBPA5 enabled the mapping of FN fiber tension in tissue cryosections from healthy mouse and human organs, as well as naturally occurring and induced cancer models [37], [38], [44], [45], revealing that large fractions of tumor FN fibers are untensed compared with the predominantly highly tensed fibers in healthy organs [44]. Untensed FN fibers spatially associate with aligned, dense collagen bundles, which suggests one mechanism by which FN fibers are shielded from re-tensioning by cellular traction forces [39], [44], [45], in addition to other mechanisms, including proteolytic cleavage [29]. Increased FnBPA5 binding has likewise been observed in other pathological contexts, including virus-infected lymph nodes and microthrombi [40], [44].

In this study, we aimed to map the tensional state of FN fibers in fibrotic lung tissue cryosections using the bleomycin mouse model. Using the mechanosensitive tension probe FnBPA5, we demonstrate that the onset and progression of fibrosis in this model correlated with a reduction in FN fiber tension and that relaxed FN fibers were located proximally to thick collagen bundles. Then, building on established *in vitro* models of progressive fibrosis that rely on TGF-β1 stimulation of lung fibroblasts [46], [47], we investigated whether this mechanical signature can be recapitulated in *de novo* assembled ECM deposited by disease-relevant cells, with the goal of developing a translational system. We show that fibrosis induction, validated through established molecular biomarkers at both the mRNA and protein levels, led to a progressive loss of FN fiber tension *in vitro*, as evidenced by increased FnBPA5 binding. Finally, we tested whether drugs with reported antifibrotic activity, as indicated by reduced biomarker expression, can restore FN fiber tension to its non-fibrotic, tensed state. Collectively, our findings identify FnBPA5 binding as a novel biomarker that captures the evolving ECM mechanics during the onset and progression of IPF. To date, no established *in vitro* assay systems reliably capture the mechanical dimension of ECM remodeling during fibrosis onset, despite *in vivo* data suggesting that changes in FN fiber tension are tightly linked to disease initiation. The ability to monitor and quantify such mechanical transitions in our assay system offers a unique opportunity to develop translational, disease-relevant platforms for drug screening and testing during preclinical drug discovery.

## Results

### Fibronectin fiber tension is reduced in fibrotic lungs of bleomycin-treated mice

**Fig. 1:**
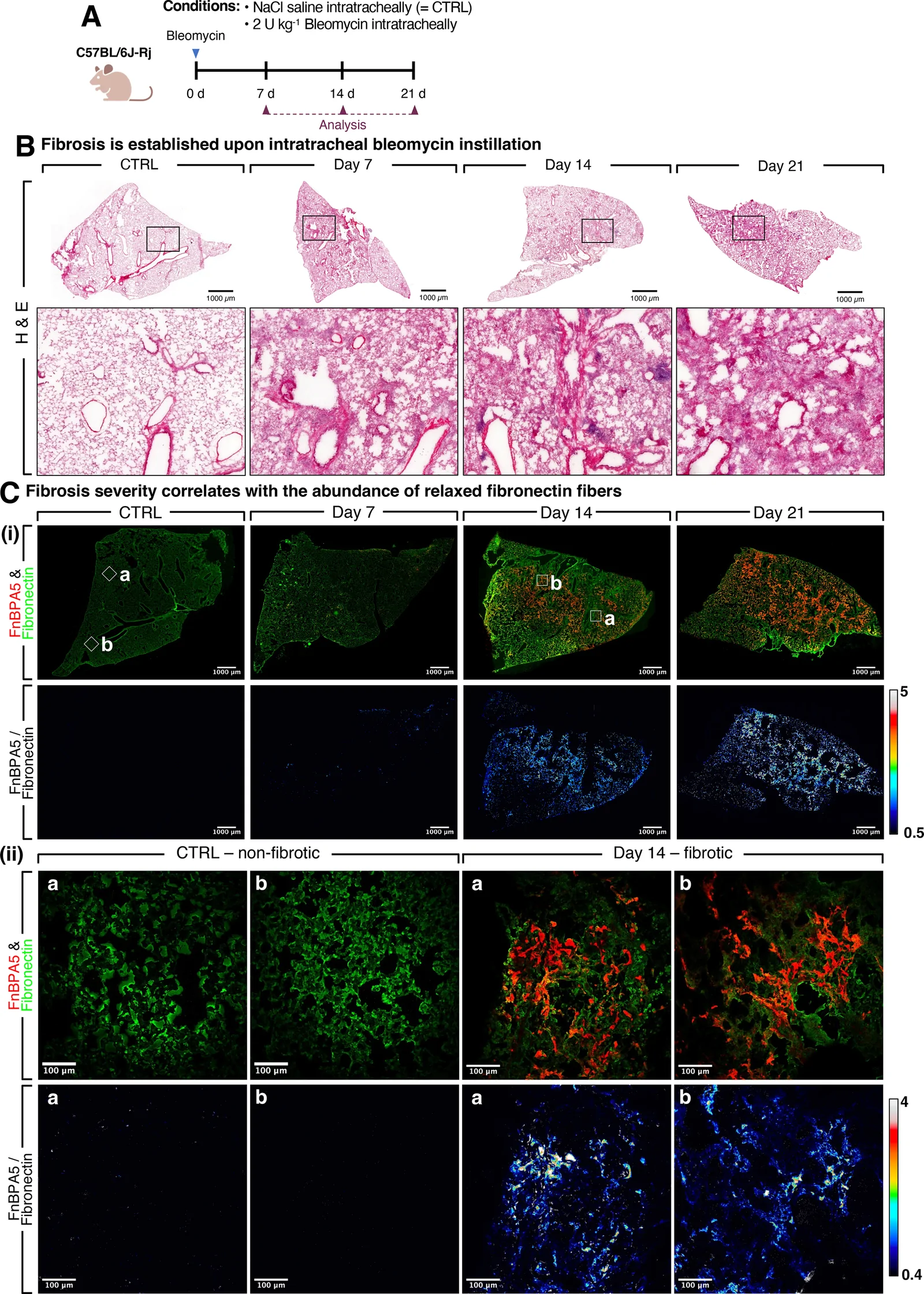
**Fibrotic lungs in the murine bleomycin model are enriched in relaxed fibronectin fibers. (A) Experimental setup.** Mice were allocated to four treatment groups: CTRL (intratracheal PBS, euthanized on day 21), and bleomycin-treated groups euthanized on day 7, day 14, or day 21 after intratracheal bleomycin administration (2.0 U kg^−1^) on day 0. **(B) Fibrosis is established upon intratracheal bleomycin instillation.** Hematoxylin and eosin images of left lung lobes illustrate changes in ECM deposition across treatment groups that indicates fibrosis induction. Successful fibrosis induction upon bleomycin was further verified by quantifying additional established biomarkers (see Fig. S2). **(C) Fibrosis severity correlates with the abundance of relaxed fibronectin fibers. (i, upper row)** Representative images of tissue cryosections showing total fibronectin (green, detected with a polyclonal anti-fibronectin antibody) and relaxed fibronectin fibers detected with Cy5-labeled FnBPA5 (red), shown for all treatment groups (CTRL; days 7, 14 and 21). Cryosections of left lung lobes were acquired as whole-tissue overview images on a 3DHISTECH Pannoramic 250 slide scanner using a 20× air Plan Apochromat objective. **(i, lower row)** Pixel-wise ratio maps (FnBPA5 signal divided by fibronectin signal) generated using the FIJI Image Calculator “Divide” function. **(ii, upper row)** Higher-resolution two-color images from adjacent sections of CTRL and day 14 lungs, cut in parallel to the tissues in panel (i) to preserve spatial correspondence. Overview images were acquired on a Nipkow spinning disk confocal microscope with a 10× air objective (see Supplementary Fig. S5). Regions of interest (a and b) were then imaged at higher resolution on a Leica SP8 line-scanning confocal microscope using a 25× 0.95 NA HCX IRAPO L water-immersion objective; ROI locations and extents correspond to those indicated on the slide-scanner images in panel (i). **(ii, lower row)** Ratiometric images for these high-resolution ROIs were generated using the same FIJI “Divide” procedure applied to the FnBPA5 and fibronectin single-channel images. Single-channel images for both whole-tissue overviews and magnified regions are provided in Fig. S3. A second biological replicate of all treatment groups (CTRL; days 7, 14, and 21; one additional mouse per group) is shown in Fig. S4. Control stainings to assess tissue autofluorescence, non-specific primary-antibody binding (fibronectin: primary antibody omitted, only AF488-tagged secondary antibody applied), and non-specific FnBPA5 binding (scrambled control peptide with identical amino-acid composition but randomized sequence, Cy5-labeled) are presented in Fig. S5. (For interpretation of the references to color in this figure legend, the reader is referred to the web version of this article.)

First, we sought to investigate the tensional state of FN fibers in fibrotic lung cryosections using the bleomycin-induced fibrosing ILD mouse model. In our experimental setup (Fig. 1A), female C57BL/6J-Rj mice were randomly allocated into subgroups, intratracheally instilled with 2 U kg^−1^ bleomycin on day 0, and euthanized according to their assigned treatment group (1–3) on day 7 (peak inflammation; group 1), day 14 (peak fibrosis; group 2), or day 21 (late fibrosis; group 3) [48]. Control mice received saline intratracheally and were sacrificed on day 21 (group 4).

Hematoxylin and eosin (H & E) staining of cryosections from the left lung lobes indicated elevated ECM deposition on day 7 after bleomycin instillation, which further intensified at later time points, as evidenced by dense cellular aggregates and progressively remodeled ECM architecture (days 14 and 21; Fig. 1B). Ashcroft scores as semi-quantitative measures of fibrosis severity [49] were determined in right inferior lung lobes after collagen visualization with Picrosirius Red (Fig. S2Ai). They were significantly increased at all time points following bleomycin instillation compared to those in saline-treated control mice (Fig. S2Aii).

The early bleomycin-induced inflammatory response involves considerable infiltration of leukocytes (*i.e.*, macrophages, neutrophils, and lymphocytes) into the lung tissue [50], which was determined by immunohistochemical (IHC) CD45 staining. The amount of CD45-positive tissue was significantly enriched at all time points compared to healthy mice, with a peak at day 7 that decreased at later time points (Fig. S2B). Macrophage accumulation, detected by staining for F4/80 by IHC, revealed a similar pattern with significantly enriched F4/80-positive tissue at all three time points post-bleomycin instillation compared to control mice, with slightly lower values on day 21 (Fig. S2C), indicating fibrosis resolution at later time points. Expression of inflammatory markers C – C motif chemokine ligand 2 (*Ccl2*) and interleukin-6 (*Il6*) was significantly elevated at early time points (day 7 and day 14), when inflammation was at its peak, but was non-significant at day 21 due to high variability between mice, which may be attributed to non-uniformly subsidizing inflammation in individual mice (Fig. S2D).

Finally, we asked whether the onset and progression of fibrosis in murine lungs are linked to a loss of FN fiber tension. Cryosections from the left lung lobes were stained with a polyclonal FN-targeting antibody to visualize all FN fibers and isoforms, whereas untensed FN fibers were detected via N-terminal binding of FnBPA5-Cy5 (Fig. 1Ci). Single-channel grayscale images are shown in Fig. S3, and data from a second mouse per treatment group are presented in Fig. S4. At all time points, cryosections displayed an extensive FN fibril network, as expected. Regarding FN fiber strain, no FnBPA5-Cy5 signal was detected in the saline-treated controls. In bleomycin-treated mice, the FnBPA5-Cy5 signal only marginally increased on day 7 (peak inflammation, onset of fibrosis). In contrast, at days 14 and 21 (established fibrosis), the FnBPA5-Cy5 signal was pronounced throughout the lung cryosections, indicating that a substantial portion of the FN fibril network contained relaxed N-termini at peak fibrosis. To exclude tissue autofluorescence and non-specific binding of either the secondary antibody or FnBPA5, we included a set of staining controls performed on adjacent cryosections. These included (i) omission of the primary FN antibody and (ii) replacement of FnBPA5-Cy5 with Cy5-labeled scrambled peptide scr-Cy5 (same amino acid composition but different sequence). Both conditions showed no or only marginal fluorescence (Fig. S5), confirming the specificity of FnBPA5 binding. As local increases in FN expression could theoretically produce more FnBPA5 binding sites due to higher FN fiber abundance, we generated ratiometric heatmaps of the FnBPA5-Cy5 to FN signal. These revealed a clear increase in relaxed FN fibers at peak fibrosis, both across the entire sections (Fig. 1Ci, lower row) and in high-resolution blow-up views (Fig. 1Cii, lower row). Altogether, these findings demonstrate that FN fiber tension diminishes during pathological remodeling of lung architecture in this mouse model, highlighting relaxed FN fibers as a promising fingerprint of fibrotic disease severity.

### Collagen fibrils co-localize with regions of relaxed FN fibers

**Fig. 2:**
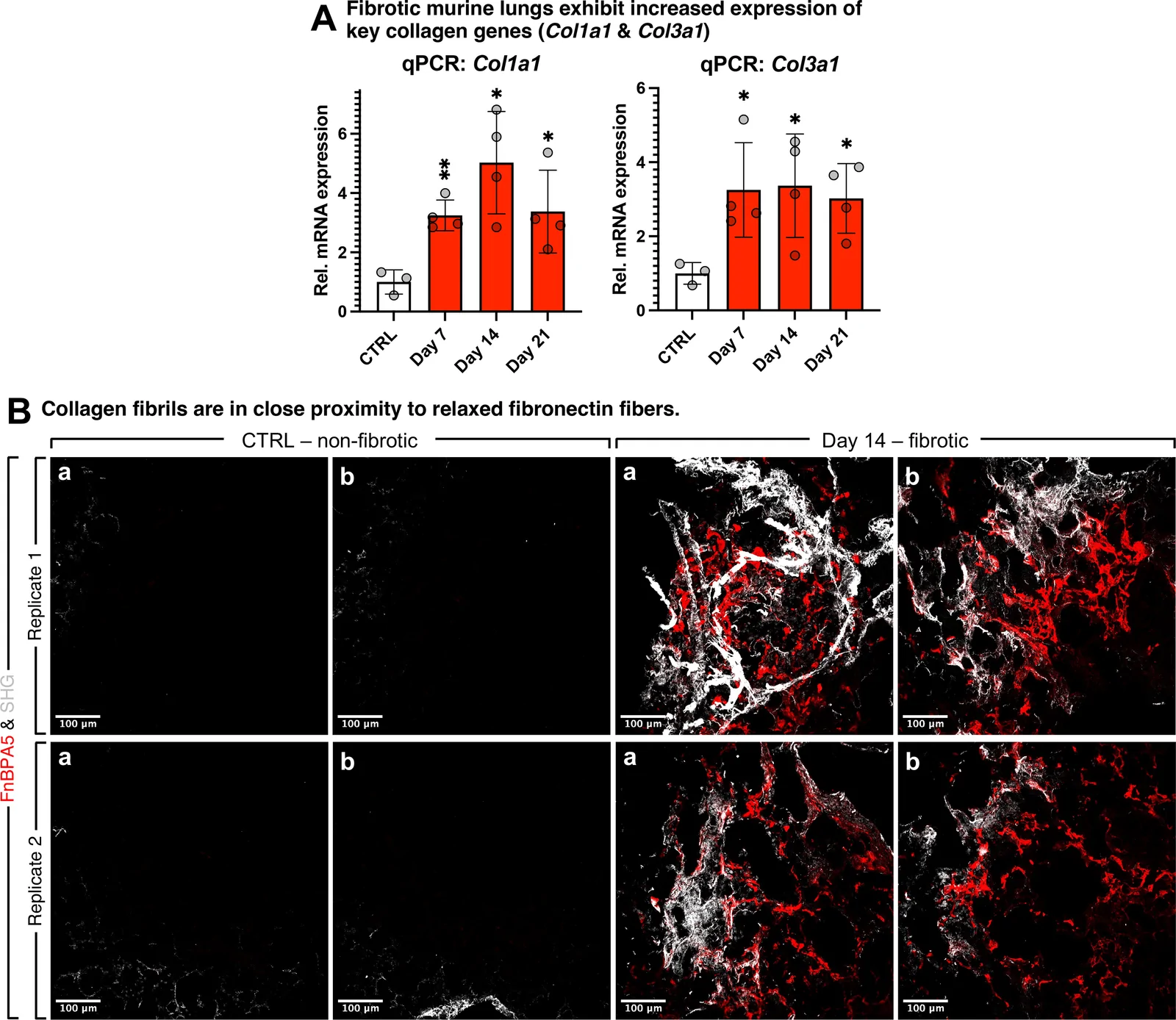
**Collagen fibrils co-localize with relaxed fibronectin fibers. (A) Fibrotic murine lungs exhibit increased expression of key collagen genes (*Col1a1* & *Col3a1*).** RT-qPCR quantification of *Col1a1* and *Col3a1* expression in right superior lung lobes. Expression values were normalized to *Rplp0*. Biological replicates: CTRL (*n* = 3 mice), and days 7, 14, and 21 post-bleomycin (each *n* = 4 mice). Data normality was assessed using the Shapiro-Wilk test; variance homogeneity using Levene's test. Statistical significance was tested using Welch's ANOVA with post-hoc Welch's *t*-tests. *P* < 0.05 was considered significant (* *p* < 0.05; ** *p* < 0.01). **(B) Collagen fibrils localize near relaxed fibronectin fibers.** Representative images of FnBPA5-positive relaxed fibronectin fibers (red) and second harmonic generation (SHG)-positive collagen fibrils (collagen I/III-containing; gray-scale) from non-fibrotic CTRL lungs and day-14 fibrotic lungs. Shown are high-resolution regions of interest (ROI a and b) acquired on a Leica SP8 line-scanning confocal microscope using a 25×, 0.95 NA HCX IRAPO L water-immersion objective. ROI positions correspond to those shown for replicate 1 in Fig. 1Ci and for replicate 2 in Fig. S5 (left panel, replicate 1; ROIs labeled as a and b accordingly). (For interpretation of the references to color in this figure legend, the reader is referred to the web version of this article.)

A hallmark of fibrotic tissue remodeling is the upregulation of several collagen types and reorganization of their extracellular architecture, resulting in tissue-level changes such as increased stiffness [51]. RT-qPCR analysis confirmed strong induction of collagen I and III (*Col1a1* and *Col3a1*) gene expression at all time points in bleomycin-treated mice compared to saline controls (Fig. 2A). Previous studies have suggested that relaxed FN fibers can act as scaffolds to induce collagen fibrillogenesis, after which collagen fibrils may reduce the tensile loading on FN [27]. To investigate whether related patterns emerge during fibrosis, we examined collagen fiber organization together with FnBPA5 binding in interstitial regions of mouse lungs after bleomycin treatment. To visualize mature collagen I and III fiber bundles, we used second harmonic generation (SHG) imaging, a label-free two-photon process sensitive to the helical structure of collagen fibrils, and imaged FnBPA5-Cy5 binding in the same regions of interest (ROI). Representative images from two mice per group are shown in Fig. 2B for non-fibrotic controls and for the peak fibrotic stage on day 14. In the control lungs, the SHG signal was negligible. In contrast, bleomycin-treated lungs showed pronounced collagen bundles on day 14. These bundles frequently appeared near areas with elevated FnBPA5 signal and shared similar orientations within the tissue. While the data suggest a spatial correlation between relaxed FN fibers and collagen bundles, further studies are required to establish a temporal correlation between the two.

### Patient-derived lung fibroblasts stimulated with TGF-β1 establish fibrotic niches *in vitro*

**Fig. 3:**
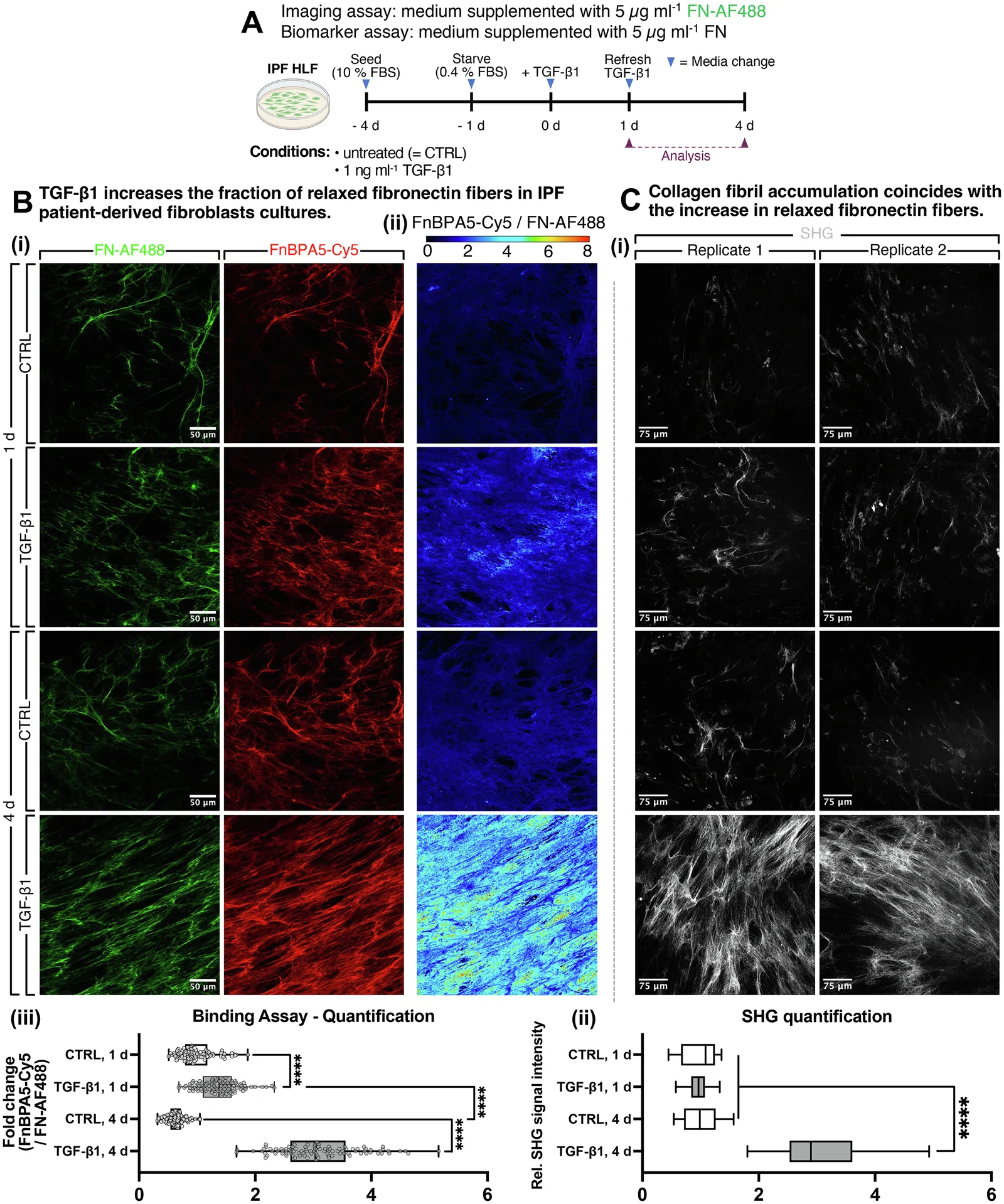
**TGF-β1–induced activation of IPF patient-derived human lung fibroblasts (HLFs) generates fibrotic *in vitro* niches with a *de novo*-assembled ECM enriched in relaxed fibronectin fibers. (A) Experimental design.** IPF-derived HLFs were seeded 3 days prior to serum starvation. After 24 h in starvation medium, cells were stimulated with 1 ng mL^−1^ TGF-β1 to induce a fibrotic phenotype. Samples were analyzed after 1 day and 4 days of TGF-β1 exposure. Successful fibroblast-to-myofibroblast transition was verified by quantifying known fibrotic markers (see Fig. S6). **(B) TGF-β1 increases the fraction of relaxed fibronectin fibers in IPF patient-derived fibroblasts cultures.** (i) The fibronectin fibrillar network was visualized by incorporation of medium-supplemented AF488-labeled fibronectin during ECM assembly. Fiber strain was assessed at endpoint using the fibronectin tension probe FnBPA5-Cy5. (ii) Pixel-wise ratiometric heatmaps were generated to display FnBPA5 binding relative to total labeled fibronectin content. (iii) Quantification of FnBPA5-Cy5 / FN-AF488 ratios from *n* = 96 imaging sites per condition (8 ROIs per well, 3 wells per experiment, 4 independent experiments; 1 donor). Imaging was performed on a Leica SP8 line-scanning confocal microscope (25×, 0.95 NA HCX IRAPO L water-immersion objective) in single-photon confocal mode. Specificity of FnBPA5-Cy5 for relaxed fibronectin fibers was confirmed by staining matched samples with the scr-Cy5 control peptide using the same staining protocol, which yielded no detectable scr-Cy5 binding (Fig. S7). The TGF-β1-induced increase in relaxed fibronectin fibers was further validated in a second IPF cell donor (Figs. S8 & S11). **(C) Collagen fibril accumulation coincides with the increase in relaxed fibronectin fibers.** (i) Collagen I/III fibrils were visualized by second harmonic generation (SHG) using the same Leica SP8 platform operated in two-photon SHG excitation mode (25×, 0.95 NA HCX IRAPO L water-immersion objective). Two representative images are shown per condition. (ii) SHG intensity per frame was quantified and summarized as box plots. Data represent three wells per condition from one experimental round, with four ROIs imaged per well. **Statistics.** Box plots: center line = median; box = 25th–75th percentile; whiskers = min-max. Normality was assessed by Shapiro-Wilk test and variance homogeneity by Levene's test. For normally distributed, homoscedastic data (C, ii), significance was evaluated using unpaired two-sided multiple-comparison *t*-tests with Benjamini-Hochberg correction. For non-normal data (B, iii), significance was assessed using pairwise Wilcoxon rank-sum tests with Benjamini-Hochberg correction. *P* < 0.05 was considered significant (* *p* < 0.05; ** *p* < 0.01; *** *p* < 0.001; **** *p* < 0.0001).

To investigate whether the loss of FN fiber tension observed during early lung fibrosis *in vivo* can be recapitulated *in vitro*, we established a 2D phenotypic imaging assay using primary human lung fibroblasts (HLF) from patients with IPF (Fig. 3A). HLFs incorporate exogenously supplemented dimeric FN into their self-assembled ECM [27], [52], [53], enabling the visualization of the developing network by adding AF488-labeled FN (FN-AF488) to the culture medium. Fibrotic activation was induced by TGF-β1, which triggers a fibroblast-to-myofibroblast transition (FMT) characterized by the emergence of α-SMA–positive stress fibers in cells [54]. We monitored FMT progression on days 1 and 4 after TGF-β1 stimulation and assessed how these changes were related to the loss of FN fiber tension.

TGF-β1 induced the transition of fibroblasts into myofibroblasts, which led to a significant upregulation of *ACTA2*, the gene encoding α-SMA, as measured by RT-qPCR (Fig. S6Ai). Correspondingly, α-SMA-positive stress fibers were clearly detectable on days 1 and 4, as validated by western blotting and immunofluorescence microscopy (Fig. S6Aii, iii). In addition to *ACTA2*, other fibrosis-associated genes, including *FN1* (fibronectin), *COL1A1* (collagen I), and *COL3A1* (collagen III) [55], [56], [57] were examined using RT-qPCR (Fig. S6Bi). *FN1* and *COL1A1* were already significantly increased after 1 day of TGF-β1 treatment, and all three genes were strongly upregulated after 4 days of treatment. To further assess collagen synthesis at the protein level, collagen I pro-peptide α1 was quantified in the cell culture supernatants by ELISA (Fig. S6Bii). As this N-terminal pro-peptide is cleaved in equimolar amounts during collagen I maturation, its concentration reflects the rate of collagen synthesis. Pro-peptide α1 levels remained unchanged after 1 day of TGF-β1 treatment but were significantly elevated on day 4. Together, these results confirm the successful induction of a fibrotic niche in our *in vitro* assay system.

### The emerging fibrotic phenotype *in vitro* correlates with a progressive loss of fibronectin fiber tension

To investigate whether FMT in our *in vitro* system leads to a loss of FN fiber tension, we used FnBPA5-Cy5 to visualize relaxed FN fibers. Upon TGF-β1 stimulation, the FN fiber network became denser, the FN-AF488 signal increased slightly, and fibers appeared thinner and more aligned, particularly on day 4 (Fig. 3Bi). FnBPA5-Cy5 binding was low in control cultures but increased markedly after TGF-β1 treatment, with the strongest signal observed on day 4 (Fig. 3Bi). To highlight regions enriched in relaxed FN fibers, we generated ratiometric heatmaps of FnBPA5-Cy5 relative to FN-AF488. These maps revealed a clear color shift after day 1 of TGF-β1 stimulation, which intensified by day 4 (Fig. 3Bii). Quantification confirmed a significant increase in relaxed FN fibers: the FnBPA5-Cy5/FN-AF488 ratio rose 1.5-fold by day 1 and up to 3-fold by day 4 (Fig. 3Biii). These mechanical changes closely followed the induction of fibrotic biomarkers (Fig. S6). Binding specificity was confirmed using a scrambled control peptide, which showed minimal non-specific binding in both control and TGF-β1-treated cultures (Fig. S7).

As a control experiment to examine whether plasma and cellular FN isoforms are equally distributed within FN fibers in our assay system, we supplemented the medium with plasma FN-AF488 as before and stained cellular FN with an antibody targeting the EDA domain, exclusively found in cellular FN (Fig. S8). Under untreated conditions (= CTRL), both isoforms displayed similar patterns consistent with their expected colocalization in FN fibers (Fig. S8A). Following TGF-β1 treatment, however, the two isoforms showed slightly distinct distributions, which were more apparent in the heatmaps that show the difference between the two signals (Fig. S8B). The FnBPA5-Cy5 signal closely matched the pattern of exogenous plasma FN (FN-AF488) when TGF-β1 was present (Fig. S8C), suggesting that the ratio of cellular to plasma FN per fiber may shift upon TGF-β1 treatment. While TGF-β1 upregulates endogenous *FN1* (Fig. S6Bi), relaxed FN fibers appear to be primarily plasma-derived in our assay (Fig. S8C). It should be noted, however, that plasma and cellular FN share identical N-termini – where the binding epitope of FnBPA5 is located [37] – meaning the peptide cannot discriminate between the two isoforms.

To determine whether areas with relaxed FN fibers correlate with collagen fiber deposition, as observed in *ex vivo* cryosections, we performed SHG imaging in the *in vitro* assay (Fig. 3C). After 1 day of TGF-β1 stimulation, no differences in SHG-positive collagen fibers were detected compared to controls. By day 4, however, thick bundles of SHG-positive collagen fibers had formed, displaying a higher degree of alignment, mirroring the increased alignment observed in the FN fiber network. Taken together, these *in vitro* findings closely recapitulated the trends seen in the *ex vivo* stained cryosections.

### Resolving the fibroblast-to-myofibroblast transition (FMT) upon treatment with known antifibrotics across multi-parametric readouts

We then sought to understand whether fibrotic progression associated with the loss of FN fiber tension could be reversed upon treatment with antifibrotic drugs *in vitro*. We selected five drugs known to target different factors within the signaling pathways involved in IPF pathogenesis: iALK5 (ALK5 Inhibitor VIII, SB-525334), metformin, quercetin (Q), nintedanib, and dasatinib (D) (Fig. S9). iALK5 [58] directly inhibits the type I TGF-β1 receptor kinase (TGF-βRI), while reduced expression of fibrotic genes, such as *COL1A1*, has been reported upon metformin treatment through AMPK activation [46], or by inducing a trans-differentiation of myofibroblasts into lipo-fibroblasts [46], [59]. Quercetin (Q) [60] can induce consumption of the serum trace compound heme [61], which triggers carbon monoxide formation known to reduce ECM gene expression (*COL1A1*, *COL3A1* and *FN1*) by regulating HO-1 either directly or through NRF2. Quercetin also reduces *TGFBR2* expression, a subunit of the TGF-β1 receptor, through microRNA-145-mediated RNA interference [62]. In contrast, Nintedanib [63] inhibits receptor tyrosine kinases, such as the PDGF, FGF, and VEGF receptors, which are crucial for fibrotic progression. Nintedanib and dasatinib inhibit cellular Src [64], [65], [66]. Dasatinib (D) treatment is known to reduce α-SMA/*ACTA2* and Fibronectin/*FN1* expression through a putative Src/SRF pathway [67]. As a combinatorial treatment, Q + D was reported to deplete senescent cells from bleomycin-instilled fibrotic murine lungs [68] and significantly improved health parameters of physical function in a pilot human study with IPF patients [69].

Prior to assessing whether drug treatments restored FN fiber tension, we validated treatment effects using fibrotic biomarkers. Cells were seeded, serum-starved, activated by TGF-β1, and treated with antifibrotic drugs, as summarized in Fig. S10A. The expression levels of *ACTA2*, *COL1A1*, *COL3A1*, and *FN1* were analyzed by RT-qPCR, collagen I pro-peptide α1 was measured by ELISA, and α-SMA was quantified by western blotting. Finally, we monitored the impact of drug treatments on cell viability to ensure that biomarker and FN fiber tension data were not confounded by drug-induced cytotoxicity.

The expression levels of *ACTA2* increased 23-fold when HLF cells were exposed to TGF-β1 and decreased significantly (to 6-fold for iALK5, 12-fold for metformin, 13-fold for quercetin, 7-fold for dasatinib, and 5-fold for Q + D) upon drug treatment, except for nintedanib, which significantly increased to 32-fold (Fig. S10Bi). In line with *ACTA2* mRNA levels, α-SMA protein levels in western blots clearly increased when treated with TGF-β1 for 4 days; however, decreases in band intensities were only visible upon treatment with iALK5, quercetin, and Q + D (Fig. S10Bii). Densitometric analyses of α-SMA/GAPDH protein ratios revealed similar trends for quercetin and Q + D, with a drop from 3.1-fold upon TGF-β1 alone to 1.8-fold upon drug treatment.

*COL1A1* and *COL3A1* expression significantly increased when cells were treated with TGF-β1 (to 2.5-fold for *COL1A1* & 2-fold for *COL3A1*) and decreased significantly when treated with iALK5 (to 1-fold for *COL1A1* & *COL3A1*) or nintedanib (to 1.5-fold for *COL1A1* & 1-fold for *COL3A1*; Fig. S10Ci). In contrast, *COL1A1* and *COL3A1* expression levels remained unchanged upon treatment with metformin, quercetin, or Q + D. Notably, *FN1* expression dropped significantly from a 7-fold increase upon TGF-β1 treatment to a 4-fold and 2.5-fold increase when treated with nintedanib and iALK5, respectively, while quercetin and metformin did not induce significant changes.

Quantification of soluble collagen I pro-peptide α1 in cell culture supernatants by ELISA revealed trends identical to those observed with *COL1A1* mRNA expression (Fig. S10Cii). Pro-peptide α1 levels significantly increased from 300 ng mL^−1^ to ∼1200 ng mL^−1^ when exposed to TGF-β1 and dropped with Q + D, iALK5 or quercetin. Treatment with nintedanib, metformin or dasatinib alone did not significantly change pro-peptide α1 levels in supernatants.

To evaluate drug-induced cytotoxicity, which may confound data from fibrotic biomarkers and FN fiber tension readouts, overall cytotoxicity was measured by lactate dehydrogenase (LDH) release and alamarBlue assays (Fig. S10D). Relative cell viabilities, as determined by the LDH assay, remained mostly unchanged for all conditions, suggesting that the drugs used did not confound biomarker data through cytotoxicity. However, we observed a noticeable decline in relative resorufin fluorescence in alamarBlue assays, with values of ∼45% for Q + D, ∼55% for metformin, ∼70% for dasatinib, ∼80% for quercetin, and ∼90% for nintedanib compared to the TGF-β1 control (=100%), indicating that the cells experience a mild metabolic burden.

### Restoration of fibronectin fiber tension following antifibrotic drug treatment

**Fig. 4:**
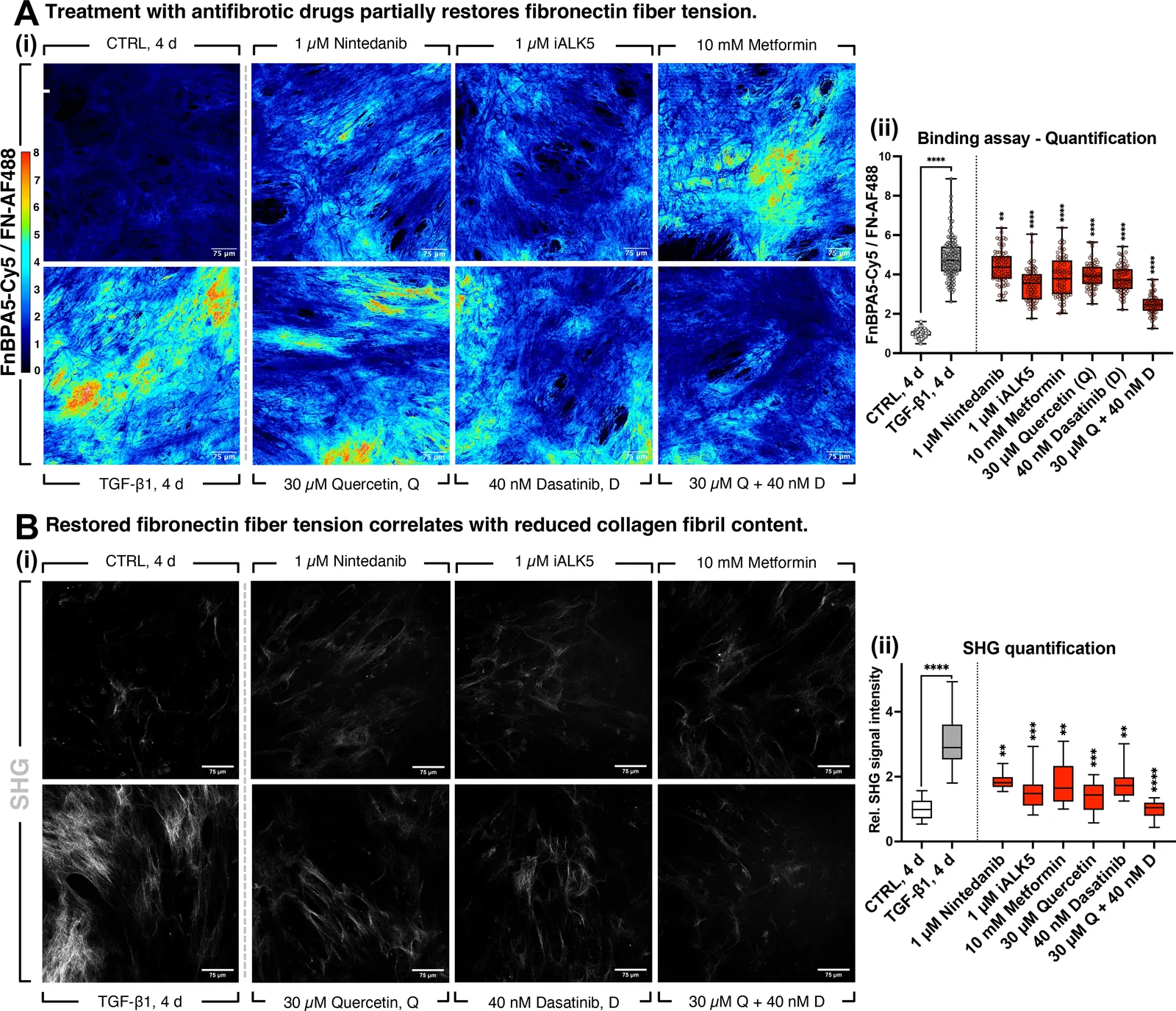
**TGF-β1–induced relaxation of FN fibers in the *in vitro assay* is reversed by antifibrotic drugs and correlates with reduced SHG-positive collagen fibrils. (A) Antifibrotic drug treatment partially restores FN fiber tension.** IPF fibroblasts were seeded and cultured as in Fig. 3. After 3 days of drug treatment, cells were incubated with 5 μg mL^−1^ FnBPA5-Cy5 to report the tensional state of FN fibers. Imaging was performed on a Leica SP8 line-scanning confocal microscope (25×, 0.95 NA, HCX IRAPO L water-immersion) in single-photon mode. FN tension was quantified by pixel-wise FnBPA5/FN-AF488 intensity ratios. (i) Representative heatmaps showing a single z-layer per condition with per-pixel FnBPA5/FN-AF488 ratios. (ii) Quantification of FnBPA5/FN-AF488 ratios. Each datapoint represents the mean ratio across the full z-stack for a single ROI. Data include 3 independent experimental rounds for each drug condition and 5 rounds for controls (CTRL and TGF-β1), with cells seeded in three wells per round. For each sample, 8 ROIs were acquired as z-stacks with 3 z-layers, yielding *n* = 120 for CTRL and TGF-β1 and *n* = 72 for each antifibrotic treatment. See Fig. S11 for the same data trends with an additional IPF cell donor. **(B) Restoration of FN fiber tension correlates with reduced collagen fibril content.** Following the same treatments as in (A), collagen I/III fibrils were imaged by SHG using the same microscope in two-photon mode. Cells from donor 1 were seeded in three wells per condition, and four ROIs were imaged per well (total *n* = 12). (i) Representative single-channel SHG images for each condition. (ii) Summary of all ROIs displayed as box plots. **Statistics.** Normality was assessed using the Shapiro–Wilk test and variance homogeneity using Levene's test for all datasets. For (Aii), FnBPA5/FN-AF488 ratios were non-normally distributed and were therefore compared against the corresponding TGF-β1 control using pairwise Wilcoxon rank-sum tests with Benjamini–Hochberg correction. For (Bii), due to unequal variances, Welch's *t*-test was applied with Benjamini–Hochberg correction for multiple comparisons. Box-plot conventions (Aii, Bii): midlines indicate medians; boxes, 25th–75th percentiles; whiskers, min–max. *P* < 0.05 was considered significant (*p* < 0.05; ** *p* < 0.01; *** *p* < 0.001; **** *p* < 0.0001).

To study the effect of antifibrotic drugs on FN fiber tension in the *in vitro* assay, we quantified the pixels with bound peptide FnBPA5-Cy5 versus AF488-labeled FN fibers, as described earlier, shown as ratiometric heatmaps (Fig. 4Ai).

TGF-β1 increased total FN fiber deposition, as observed by a denser fiber network, but also significantly increased the fraction of relaxed FN fibers, as determined by the FnBPA5-Cy5/FN-AF488 ratio (=1 for CTRL, ∼5 for TGF-β1). Treatment with iALK5 or Q + D resulted in a visible reduction in bound FnBPA5-Cy5 when qualitatively examining the heatmaps, indicating an increase in FN fiber strain, in line with the biomarker data in Fig. S10. Statistical analysis of all replicates and imaged ROIs revealed significantly reduced peptide binding for all added drugs compared to the respective TGF-β1 condition (Fig. 4Aii). Binding ratios decreased from ∼5 for TGF-β1 down to ∼2.5 for Q + D (**** *p* ≤ 0.0001) and to ∼3.5 for iALK5 (**** *p* ≤ 0.0001). Decreases in peptide binding for nintedanib, metformin, quercetin and dasatinib (only) were less pronounced with values around ∼4.5 for nintedanib (** *p* ≤ 0.01) and ∼ 4 for the other treatments (all **** *p* ≤ 0.0001).

To strengthen our conclusions on FN fiber mechanics during FMT *in vitro* and how it is modulated by antifibrotic drugs, we repeated key experiments with cells from a second IPF patient. This allowed us to confirm that our findings were consistent across donors and not patient-specific. Cells were stimulated with TGF-β1 as before and then treated with either iALK5 or nintedanib for three consecutive cycles for three days each, with fresh drug added at each treatment point (see Fig. S11A). After the third treatment, we quantified the FnBPA5-Cy5 to FN-AF488 ratio. Data from cell donor 2 mirrored our initial results: TGF-β1 treatment led to a clear reduction in FN fiber tension *in vitro*, and both antifibrotic drugs, iALK5 and nintedanib, restored FN fiber tension (Fig. S11B). We related these mechanical alterations to increased collagen fiber deposition by measuring collagen I pro-peptide α1 concentrations in cell culture supernatants via ELISA (Fig. S11C), as previously done for cell donor 1 (Fig. S10Cii). Together, these results show that the negative correlation between the loss of FN fiber tension and collagen production is reproducible across multiple cell donors.

Since these profiles were consistent across both cell donors, we next investigated whether the drug-induced alterations in the FnBPA5-Cy5 to FN-AF488 ratio (Fig. 4A) were mirrored by changes in the abundance of mature collagen fibrils (Fig. 4B). In line with the pro-peptide α1 concentrations measured *via* ELISA (Figs. S10Cii and S11C), relative SHG signal intensities were significantly lower following Q + D, iALK5, quercetin, or nintedanib administration compared to the TGF-β1-treated control; even metformin and dasatinib treatment resulted in a significant decline in SHG signal (Fig. 4B). Similarly, for donor 2, regions containing mature SHG-positive collagen fibrils clearly colocalized with areas exhibiting a high FnBPA5-Cy5 to FN-AF488 ratio (Fig. S12). Together, these results indicate that drug-induced reductions in the FnBPA5-Cy5 to FN-AF488 ratio are closely associated with a decreased presence of major collagen fiber depositions.

In summary, our results demonstrate that relaxed FN fibers serve as a robust mechanical marker of the fibrotic state of the ECM. Furthermore, our *in vitro* assay established that this fibrotic mechanical remodeling is reversible, as treatment with antifibrotic drugs can at least partially restore FN fiber tension to its native tensed state. Ultimately, our data highlight the potential of this *in vitro* platform as a high throughput tool to probe ECM mechanics in fibrosis, making it highly suitable for large-scale drug screening applications.

## Discussion

Here, for the first time, we mapped FN fiber tension in the context of *ex vivo* and *in vitro* IPF models using the mechanosensitive peptide probe FnBPA5. Using lung cryosections from bleomycin-treated mice, we demonstrated that fibrosis induction is associated with a decrease in FN fiber tension *in vivo* and that relaxed FN fibers are in close spatial proximity to collagen fiber bundles. A 2D *in vitro* assay utilizing human IPF patient-derived fibroblasts was developed, where progressively built-up fibrotic matrix was observed after 4 days of TGF-β1 stimulation, as monitored by a panel of established markers at the gene expression and protein levels and ECM architecture. This *in vitro* assay, devoid of immune cells, was able to recapitulate the relaxation of FN fibers as fibrotic remodeling advanced. Fibrosis was successfully modulated using a panel of drugs with reported antifibrotic activity. Resolving fibrosis, as observed in improved biomarker data, correlated with changes in FN fiber strain. In both models, *in vivo* and *in vitro*, ongoing fibrosis progressively reduced FN fiber strain, as indicated by increased FnBPA5 binding. Taken together, our results demonstrate a direct connection between altered FN fiber strain and IPF-associated matrix remodeling.

Recent studies, including ours, have highlighted the central role of ECM remodeling in fibrotic diseases [11], [70], [71]. However, drug discovery and development remain hampered by a lack of multiparametric *in vitro* assays that capture ECM changes as the disease progresses. The first scar-in-a-jar study aimed to profile the full spectrum of collagen biosynthesis intermediates in an *in vitro* fibrosis model, to subsequently use this system for the evaluation of antifibrotic drug candidates [72]. A subsequent study extended this assay up to 12 days and evaluated biomarkers of ECM biosynthesis in the supernatant using ELISA in a prolonged disease setting [47]. These assays served as powerful tools for monitoring fibrotic changes *in vitro* through multivariable readouts, enabling the rapid screening of candidate drugs for antifibrotic activity. However, none of these systems captured the mechanical changes in the ECM during disease progression. The *in vitro* assay developed here aims to fill this gap and provide a disease-relevant readout for evaluating the fibrotic state of the ECM. Replacing outdated *in vitro* and animal models with limited translational potential with more refined assay systems is likely to accelerate the preclinical discovery of relevant disease biomarkers.

The loss of FN fiber strain observed in both the *in vitro* assay and tissue cryosections is, from a mechanobiological perspective, counter-intuitive. Increased tissue stiffness and myofibroblast contractility, driven by excessive ECM deposition and assembly of the intracellular α-SMA machinery, respectively, are generally thought to place ECM fibers under greater tension [73], [74]. Furthermore, collagen fiber alignment has long been associated with strain-stiffening [75], leading to the widespread assumption that tissue strain correlates with ECM fiber strain; however, this is not necessarily the case [76]. Indeed, increased alignment of ECM fibers is typically considered a hallmark of a tensed fiber state [77]. One explanation for this apparent paradox is that the collagen fiber network, rather than FN, primarily bears the load of cellular tensile forces during fibrosis. Supporting this, previous studies have shown that collagen digestion with collagenase and inhibition of collagen–FN interactions using the R1R2 peptide both result in increased FN fiber strain [27], suggesting that a stiffer collagen network can effectively shield FN fibers from tension.

Interestingly, when distinguishing between cellular and plasma FN, FnBPA5 appeared to localize preferentially to plasma FN-derived fibers under TGF-β1-treated conditions, despite the upregulation of the *FN1* gene. Plasma FN-derived fibers are present from the beginning of the assay due to FN-AF488 supplementation and therefore establish a matrix before TGF-β1-induced *FN1* upregulation increases cellular FN deposition. We speculate that this temporal difference may contribute to the preferential localization of FnBPA5 to plasma FN-derived fibers. As collagen fibrils accumulate during fibroblast activation, pre-existing plasma FN-derived fibers may already be fully incorporated into the matrix, whereas cellular FN fibers are deposited concurrently and may still be undergoing assembly. Alternatively, cellular FN fibers are assembled directly following secretion and may therefore remain physically connected to cells for longer periods than plasma FN-derived fibers, which are incorporated from the extracellular space. This prolonged cell-fiber coupling could expose cellular FN longer to contractile forces that maintain FN fibers in a more stretched state [52]. Together, these factors could contribute to differences in the tensional states of plasma and cellular FN at the time points examined here.

An alternative, currently untested, hypothesis is that increasing cell packing contributes to loss of FN fiber tension. Dense cellular aggregates may absorb part of the tissue tension, thereby reducing the load borne by FN fibers. Such densely packed tissues would resemble tumor-like cell masses, in which relaxed FN fibers have been reported [39]. However, these observations were also associated with nearby collagen fibrils, making it difficult to separate the effects of collagen deposition from cell packing. Whether dense cellular aggregates alone can cause FN fibers to relax therefore remains unknown. Notably, cells are generally considered the primary source of ECM tension [52], emphasizing that tension generation and tension offloading are likely distinct processes.

As fibrosis reflects a continuous balance between matrix deposition and degradation [47], [78], the progressive relaxation of FN fibers may also reflect active ECM remodeling, potentially driven by the elevated expression and activity of proteolytic enzymes, such as matrix metalloproteinases [29], [37]. Finally, it is also worth noting that alterations in FN fiber conformation have previously been associated with a pathological ECM state [31]. Using the scFv antibody fragment H5, altered FN fiber conformation was demonstrated in bleomycin-induced fibrosis in mice—although the precise binding epitope of H5 remains unknown, precluding direct comparison with FnBPA5.

While the data presented here indicate that FN fiber relaxation is a mechanical signature of the fibrotic ECM both *ex vivo* and *in vitro*, future studies are needed to establish the clinical relevance of our findings. This could include precision-cut lung slices from patients with IPF, which is a robust platform for biomarker evaluation in a fully disease-relevant context. Previous studies have used these slices to investigate the mechanisms of fibrogenesis [79] or to explore potential therapeutic targets in IPF [80]. Since these slices originate from patients with IPF, they capture the heterogeneity of the disease more effectively than the bleomycin model [81].

Beyond IPF, key pathological mechanisms are shared with other types of progressive fibrotic interstitial lung diseases (PF-ILD). Nonspecific interstitial pneumonia, rheumatoid arthritis-ILD, and systemic sclerosis-ILD often demonstrate dysregulated TGF-β1 signaling, FMT, and aberrant production of ECM, including collagens and FN [82]. Future studies could address whether loss of FN fiber strain is a mechanical signature in these diseases as well or a method to discriminate between these indications, further extending the relevance of our approach.

Although the *in vitro* assay developed here can be used to map mechanical changes in the ECM as fibrosis progresses, further work is essential to address existing limitations. The importance of immune cell infiltration in the bleomycin-induced IPF rodent model is clear when looking at the significantly increased numbers of immune cells in the fibrotic tissue (Figs. S2B and C) at early time points. Additionally, the use of female mice only may limit the ability of this study to account for potential sex-related differences in ECM mechanics during fibrosis progression. Tissue staining with FnBPA5 has only been performed using cryosections, as it is not compatible with fixed or paraffin-embedded tissue sections, routinely used in pathology, due to epitope masking during fixation or embedding [39], therefore limiting the potential of our peptide probe as a mainstream diagnostic tool. While it is remarkable that the *in vitro* monoculture (in the absence of immune cells) recapitulates several main features of progressive fibrosis and its reversibility upon antifibrotic drugs, it does not capture the multifactorial nature of the disease arising from the dysregulated interplay of various cell types. Moreover, antifibrotic drug treatments (metformin, dasatinib and quercetin) showed discrepancies in their efficiency according to standard fibrotic readouts (Fig. S10), yet they still influenced FN fiber tension as reported by FnBPA5 binding *in vitro* (Fig. 4). It could be argued that FN fiber mechanics may reflect the overall fibrotic state of the ECM more accurately, given its position downstream of commonly used gene expression readouts.

In conclusion, we demonstrate here that loss of FN fiber tension is a mechanical signature of progressive fibrosis in *ex vivo* cryosection stains and have developed a complementary *in vitro* assay that captures hallmark features of progressive fibrosis, including the mechanical state of FN fibers. The methodology we describe offers a powerful platform for antifibrotic drug development, facilitating the screening of drug libraries to enhance translational preclinical research.

## Experimental procedures

### Animal experiments

#### Setup of *in vivo* experiments

Female C57BL/6J-Rj mice (8-week-old at experiment start) were purchased from Janvier Labs (Le Genest-Saint-Isle, France). Mice were acclimated for one week to local vivarium conditions (12 h light-dark cycle) with access to standard rodent food and water *ad libitum*. They were kept in groups of five per cage at the Laboratory Animal Service Center (LASC) at University of Zurich. Mice were allocated to groups by complete randomization. Mean body weights were monitored for each group to avoid weight-associated data bias. Fibrosing interstitial lung disease (*n* = 12) was induced by intratracheal (i.t.) instillation of 2 U kg^−1^ Bleomycin sulphate (“Baxter” 15′000 *I.E.*, Zurich Cantonal Pharmacy), dissolved in 50 μl saline, on day 0. Controls (*n* = 3) received i.t. instillation of 50 μl saline. Subsets of bleomycin-induced mice (*n* = 4) were sacrificed on days 7 (peak inflammation), day 14 (peak fibrosis/disease), and day 21 (endpoint). Controls were sacrificed on day 21. It should be noted that female mice were chosen (i) to prevent any potential sexual dimorphisms, which would give rise to high variability due to the limited sample size (*n* = 12) and (ii) out of animal welfare considerations, as male mice are known to exhibit higher disease severity and therefore higher mortality rate compared to female mice [83]. All animal experiments were approved by the cantonal authorities and performed in strict compliance with the Swiss law for animal protection (license ZH235–2018, approval number 30767).

#### Lung tissue collection

Mice were euthanized, and whole lung samples were excised and further dissected into individual lung lobes for further processing:

1. Left lung lobe: Staining of cryosections with FnBPA5-Cy5 for FN fiber strain studies; H & E.
2. Right inferior lung lobe: Picrosirius Red for Ashcroft scoring; IHC of CD45 + F4/80; H & E.
3. Right superior lung lobe: Gene expression profiling in tissues using RT-qPCR.

#### IHC stainings for FN and FN fiber tension with FnBPA5-Cy5

For the preparation of frozen tissue sections, the left lung lobe was perfused with a 1:1 mixture of PBS/O.C.T. prior to embedding and freezing in tissue cryomolds filled with O.C.T. Sections were rehydrated in Gibco™ PBS, pH 7.4 (#10010015, Thermo Fisher Scientific) and blocked in PBS + 4% BSA (#A1391, AppliChem) for 30 min. Relaxed FN fibers were detected by incubating with PBS + 5 μg mL^−1^ Cy5-labeled FnBPA5 peptide for 1 h, followed by washing 3× with PBS. Tissues were fixed with 4% formaldehyde (#P087.1, Carl Roth GmbH) for 10 min at RT and washed 6× with PBS. Prior to incubation with antibodies, the sections were blocked a second time with PBS + 5% normal goat serum (#G9023-10ML, Sigma-Aldrich) for 1 h. For FN staining in tissues, sections were incubated with an FN-targeting polyclonal antibody (#ab23750, Abcam) at a dilution of 1:100 in PBS + 5% normal goat serum ON, followed by washing 3× with PBS. After incubation with primary antibodies, the tissue sections were incubated with AF488-labeled goat-anti-rabbit secondary antibody (#A-11034, Thermo Fisher Scientific) diluted 1:200 in PBS + 5% normal goat serum for 1 h at RT, followed by washing 3× with PBS. The tissues were mounted in Prolong Gold antifade reagent (#P36930, Thermo Fisher Scientific) and sealed with nail polish when dried. Tissue sections were imaged and stitched using a Slide Scanner Pannoramic 250 3DHISTECH (Figs. 1Ci, S3A, and S4).

#### Histology and CD45 + F4/80 IHC stainings

The inferior lobe of the right lung was perfused and fixed overnight in 10% neutral buffered formalin at RT before embedding in paraffin. Formalin-fixed paraffin-embedded sections at 3 μm thickness were prepared and stained with H&E for examination of tissue architecture and cellular infiltrates, and Picrosirius Red (PSR) for visualization of collagen deposition, using standard protocols [84]. Sections were pre-treated using heat-induced epitope retrieval with citrate-based buffer (pH 6, BOND Epitope Retrieval Solution 1, ER1) or EDTA-based buffer (pH 9, BOND Epitope Retrieval Solution 2, ER2) prior to antibody incubation and detection of the target proteins. Immunostaining for CD45 and F4/80 was performed on a Leica BOND-MAX instrument (Biosystems Switzerland AG) using the BOND Polymer Refine Detection Kit (1:1000 F4/80, #70076, Cell Signaling Technology; 1:500 CD45, #550539, BD Pharmingen; BOND Primary Antibody Diluent, #AR9352, Leica). 3,3′-Diaminobenzidine (DAB) was used as the chromogen for detection, and the sections were counterstained with hematoxylin.

Complete tissue sections were imaged using an Axio Scan.Z1 (Zeiss, Feldbach, Switzerland) slide scanner in bright-field mode with a Plan-Apochromat 20×/0.8 M27 objective. IHC sections were automatically quantified using the open-source software Orbit Image Analysis (Version 3.24, Actelion Pharmaceutical Ltd) as described in [85], [86]. Histopathological quantification of fibrosing ILD was performed using Ashcroft scoring [49] on PSR-stained lung sections by two experienced and blinded examiners, as described in [87].

#### *Ex vivo* RT-qPCR gene expression profiling of murine lung tissue sections

Total RNA was isolated from the superior lobe of the right lung, preserved in RNAlater solution. Murine lung tissues were mechanically homogenized by 4 × 30 s cycles at 30 Hz with inter-cycle cooling on ice using Qiagen's TissueLyser II (#85300) and RNA was isolated using Qiagen RNeasy Mini Kit (#74104). cDNA was synthesized using the Roche Transcriptor First Strand cDNA Synthesis Kit (#04379012001). The expression levels of fibrosis (*Col1a1*, *Col3a1*, *Fn1*) and inflammation-associated (*Il6*, *Ccl2*) genes were quantified by SYBR Green-based RT-qPCR using the GoTaq qPCR Master Mix (#6001, Promega) on a 7500 Real-Time PCR System instrument (Applied Biosystems) as described in [87].

Primers were purchased from Microsynth:

1) *Rplp0* fw: GCAGGTGTTTGACAACGGCAG, rv: GATGATGGAGTGTGGCACCGA
2) *Col1a1* fw: GATGACGTGCAATGCAATGAA, rv: CCCTCGACTCCTACATCTTCTGA
3) *Col3a1* fw: AGCTTTGTGCAAAGTGGAACC, rv: ATAGGACTGACCAAGGTGGC
4) *Fn1* fw: ATGTGGACCCCTCCTGATAGT, rv: GCCCAGTGATTTCAGCAAAGG
5) *Ccl2* fw: CCACTCACCTGCTGCTACTCAT, rv: TGGTGATCCTCTTGTAGCTCTCC
6) *Il6* fw: TGATGGATGCTACCAAACTGG, rv: GGTACTCCAGAAGACCAGAG

*Rplp0* was used as a housekeeping gene for normalization. Gene expression fold changes were calculated using the 2^-ΔΔCt^ method.

### *In vitro* experiments

#### Fibronectin isolation and labeling

Fibronectin was isolated from human plasma (Blood Bank Zurich) using gelatin sepharose chromatography, as described by Speziale *et al*. [88]. Plasma was thawed and passed through a PD-10 column (#17–0851-01, GE Healthcare), and the effluent was collected and run through a gelatin sepharose column. After washing the column, FN was eluted from the gelatin column with a 1.4 M arginine-HCl solution (pH 7.4). FN was then re-buffered with PBS before use. FN aliquots were stored until further use at −80 °C. For labeling, FN was re-buffered to 0.2 M carbonate buffer (pH 8.5) (Na_2_CO_3_: #13418-1KG-R, Sigma-Aldrich; NaHCO_3_: #S5761–500G, Sigma-Aldrich). FN was labeled upon reaction with Alexa Fluor 488 NHS ester (#A20000, Thermo Fisher) for 1 h at room temperature at a 60-fold molar excess, according to the supplier's instructions. The labeled protein was then separated from the residual free dye and re-buffered in PBS by elution from a PD-10 column as described above.

#### Culturing of IPF cells

Primary, patient-derived IPF cells (#CC-7231, Lonza) were cultured in Dulbecco's Modified Eagle's Medium, low glucose, GlutaMAX™ Supplement, pyruvate (DMEM, #21885025, Gibco™ ThermoFisher Scientific), supplemented with 10% (v/v) fetal bovine serum (FBS, #S181H-50, biowest VWR) and 1% (v/v) penicillin-streptomycin (#15140122, Gibco™ ThermoFisher Scientific) in a humidified incubator containing 5% CO_2_ at 37 °C. The primary cell lines were obtained from two independent individuals. Donor 1 was a 52-year-old Caucasian male, whose cells were isolated and expanded by Lonza before cryopreservation on April 27, 2017. Donor 2 was an 83-year-old Caucasian male, with cells isolated and expanded by Lonza and cryopreserved on December 20, 2018. Cells from both donors were received at frozen passage 2 and used for fibrosis assays between passages 4 and 8.

#### Peptide binding assay

IPF cells were grown to confluence and seeded at 20,000 cells per well in fibronectin-coated (20 μg mL^−1^ FN in PBS ON at 4 °C) 8-well Nunc Lab-Tek chambers (#155411, Thermo Fisher Scientific) in high serum (10% FBS) DMEM supplemented with 100 μM l-ascorbic acid (AA, #A8960-5G, Sigma-Aldrich). Ascorbic acid was supplemented as it is essential for the post-translational modification of proline to hydroxyproline to allow for the correct folding of collagens during synthesis [47]. Cells were allowed to attach to the culture surface before exchanging the medium with high serum DMEM + AA supplemented with 5 μg mL^−1^ AF488-labeled FN (FN-AF488) for the assembly of cell-derived, fluorescently labeled ECM. After 72 h, high-serum DMEM was exchanged for low-serum DMEM (0.4% FBS) + AA + 5 μg mL^−1^ FN-AF488 and the cells were serum-starved for 24 h. After serum starvation, the cells were treated with TGF-β1 by medium exchange to low serum DMEM + AA + 5 μg mL^−1^ FN-AF488 supplemented with 1 ng mL^−1^ TGF-β1 (#100-21C, Peprotech). FN tensional states were probed after 1 day of TGF-β1 supplementation or 4 days, which included a 72 h treatment with antifibrotic substances. The media of day 4 samples were refreshed with media with or without TGF-β1 and further treated with 1 μM iALK5 (#SB525334, MedChemExpress), 1 μM nintedanib (Boehringer-Ingelheim), 10 mM metformin (#PHR1084-500MG, Sigma-Aldrich), 30 μM quercetin (#Q4951-10G, Sigma-Aldrich), 40 nM dasatinib (#SML2589-50MG, Sigma-Aldric), or a combination of 30 μM quercetin + 40 nM dasatinib.

For harvest at day 1 (with/without 24 h TGF-β1) or day 4 (with/without 24 h TGF-β1 + 72 h TGF-β1/treatments), respectively, cells were washed with PBS prior to incubation with serum + fibronectin-free DMEM + AA supplemented with 5 μg mL^−1^ FnBPA5-Cy5 or non-specific scr-Cy5 (both purchased from Peptide Speciality Laboratory, sequences previously described [28]) for 1 h at 37 °C. Cells were washed 3× with PBS, fixed with 4% formaldehyde (ROTI®Histofix 4%, #P087.1, Carl Roth) for 10 min, washed again 3× with PBS, and stored at 4 °C until imaging. For samples that required nuclei visualization, PBS containing 2 μg mL^−1^ DAPI (#D9564, Sigma-Aldrich) was added, followed by washing with 3× PBS before storing at 4 °C.

#### Determining expression levels of fibrotic markers with RT-qPCR

IPF cells were seeded and cultured as described above, this time in 6-well plates (#92406, TPP), while maintaining the same seeding density per area. The media were supplemented with 5 μg mL^−1^ unlabeled FN. On days 1 and 4, cells were harvested by adding 200 μl TRIzol™ Reagent (#15596026, ThermoFisher Scientific) and mechanically detached by scraping. RNA was then isolated according to the manufacturer's protocol. A total of 500 ng RNA per condition was reverse transcribed into cDNA using the iScript Advanced cDNA Kit (#172-5038, BioRad) according to the manufacturer's instructions. cDNA was diluted 1:10 in RNase-free H_2_O, and gene expression was analyzed by RT-qPCR in technical triplicates using SYBR master mix (#1725271, BioRad).

Primers were purchased from Microsynth:

1) *RPLP0* fw: ACACTGGTCTCGGACCTGAGAA, rv: AGCTGCACATCACTCAGAATTTCA
2) *ACTA2* fw: GACAATGGCTCTGGGCTCTGTAA, rv: ATGCCATGTTCTATCGGGTACTT
3) *COL1A1* fw: CAGCCGCTTCACCTACAGC, rv: TTTTGTATTCAATCACTGTCTTGCC
4) *COL3A1* fw: GGAGCTGGCTACTTCTCGC, rv: GGGAACATCCTCCTTCAACAG
5) *FN1* fw: CGGTGGCTGTCAGTCAAAG, rv: AAACCTCGGCTTCCTCCATAA

*RPLP0* was used as a housekeeping gene for normalization. Gene expression fold changes were calculated using the 2^-ΔΔCt^ method.

### Determining protein levels of fibrotic markers by Western Blotting

#### Cell harvest and lysis

IPF cells were seeded, cultured, and treated as described for the RT-qPCR samples. On days 1 and 4, respectively, cells were harvested by adding 50 μl lysis buffer (1% Triton-X-100, #437002A, VWR; cOmplete™ Protease Inhibitor Cocktail, #11836170001, Sigma-Aldrich; 40 mM HEPES, #3724, Applichem; 5 mM MgCl_2_, #63068-250G, Sigma-Aldrich; 10 mM KCl, #60130-250G, Sigma-Aldrich) to the cell layer. Cells were mechanically detached, and the lysate was homogenized by pipetting and sonication (10 pulses each 1 s; sonicator probe 102 (CE), power station Branson 550, Branson Ultrasonics) and subsequently inverted in an end-over-end shaker for 30 min at 4 °C to complete lysis. The lysate was pre-cleared by centrifugation (14,000 ×*g*, 15 min, 4 °C), and the purified supernatant was harvested for further use.

#### SDS-PAGE and wet transfer

Protein concentrations in the cell lysates were determined using the Bradford assay. Lysates were heat-denatured by incubation at 95 °C for 5 min. Proteins were separated by SDS-PAGE (running buffer prepared as 10×: 1 l H_2_O + 30 g Trizma® base, #T1503-1KG, Sigma-Aldrich; + 144 g Glycine, #G7126-1KG, Sigma-Aldrich; + 10 g SDS, #A4159, Applichem) and transferred onto Immun-Blot® PVDF Membranes (#162-0177, BioRad) by wet transfer (transfer buffer: 1.8 l H_2_O + 200 ml ethanol abs. + 4.44 g CAPS, #C2632-100G, Sigma-Aldrich, + 10 tablets NaOH, #28244.295, VWR).

#### Incubation with primary and secondary antibody and quantification

After wet transfer, membranes were blocked by immersion in 5% (m/v) milk (Rapilait, #L30195806, Migros) diluted in TBST (prepared as 10×: 1 L H_2_O + 24 g Trizma® base; + 88 g NaCl, #S5886-500G, Sigma-Aldrich; + 1% (v/v) Tween20, #P1379-500ML, Sigma-Aldrich) for 15 min. Membranes were probed with primary antibodies (1:1000 α-SMA, #ab7817, Abcam; 1:500 GAPDH, #G9545, Sigma-Aldrich; diluted in 5% (m/v) BSA, #05470-5G, Sigma-Aldrich, in H_2_O + 0.02% (m/v) sodium azide, #71290-100G, Sigma-Aldrich) while rocking at 4 °C ON. The membranes were washed 3× with TBST. The membranes were then probed with secondary antibody (1:5000 Peroxidase AffiniPure Donkey Anti-Rabbit IgG, #711–035-152 for GAPDH, Peroxidase AffiniPure Donkey Anti-Mouse IgG, #715–035-150 for α-SMA; both Jackson ImmunoResearch; in 20 ml 5% (v/v) milk in TBST for 1 h at RT on a rocker plate. The membranes were washed again as described above. Western blots were treated with WesternBright™ ECL substrate (#K-12045-C20, Advansta) according to the manufacturer's instructions and analyzed using a Fusion FX (Vilber) chemiluminescence readout system.

#### Quantifying cell viability: LDH and alamarBlue

Overall cytotoxicity was measured using lactate dehydrogenase (LDH) release (#C20300, Thermo Fisher Scientific) and alamarBlue (#DAL1025, Thermo Fisher Scientific) assays, according to the manufacturer's instructions. IPF cells were seeded as described above for RT-qPCR and WB samples while scaling to a smaller culture surface/volume: 8,000 cells per well of a black, flat-bottom, polystyrene 96-well plate (#655097, Greiner-Bio) at a total media volume of 200 μl. On the day of harvest, 50 μl of culture supernatant was transferred into a transparent, flat-bottom 96-well microtiter plate (#Z707902, TPP) to measure cytotoxicity using the LDH assay. We further removed 60 μl of medium from the original culture plate to measure cell health using the alamarBlue assay with the remaining 90 μl of culture supernatant. The absorbance and fluorescence intensities for both assays were recorded using a TECAN Spark 10 M UV/VIS plate photometer.

**AlamarBlue assay:** Relative cell viability was calculated by normalizing to the average, background-corrected A_570nm_ of the day 4 TGF-β1 sample (= 100% viability).

**LDH release assay:** Relative cell viability was determined using Eq. 1. In this equation, the day 4 TGF-β1 condition was set to 100%.

(1) $$\mathrm{Cell viability},\%=100-100\times \frac{\mathrm{LDH}\;{\mathrm{activity}}_{\mathrm{compound}}-\mathrm{mean}\left(\mathrm{LDH}\;{\mathrm{activity}}_{\mathrm{TGF\beta}1}\right)}{\mathrm{mean}\left(\mathrm{LDH}\;{\mathrm{activity}}_{\max\left(\mathrm{TGF\beta}1\right)}\right)-\mathrm{mean}\left(\mathrm{LDH}\;{\mathrm{activity}}_{\mathrm{TGF\beta}1}\right)}$$

#### Determining protein levels of fibrotic markers by ELISA

To estimate collagen synthesis in the untreated and fibrotic controls as well as in drug-treated samples, collagen I pro-peptide α1 concentrations were quantified in the cell culture supernatants. Supernatants were collected on days 1 and 4 during the peptide binding assay, before the peptide incubation step, and stored at −80 °C until further use. Collagen I pro-peptide α1 concentrations were determined according to the manufacturer's instructions (Human Pro-Collagen 1α1 DuoSet ELISA #DY6220-05 + DuoSet ELISA Ancillary Reagent Kit 2 #DY008, R&D Systems). Each sample was measured in technical duplicates. Absorbance was recorded using a TECAN Spark 10 M UV/VIS plate photometer.

#### Immunostaining of *in vitro* assay samples

Immunostaining for α-SMA or EDA/EDB-FN was performed on fixed cell samples.

To visualize α-SMA stress fibers in IPF cells, samples that had been previously stained with Cy5-labeled FnBPA5 and FN-AF488 were used, as the corresponding fluorescence channels remained available. Samples were rinsed three times with pre-warmed PBS and fixed with 4% paraformaldehyde for 10 min, followed by three PBS washes. Cells were permeabilized for 20 min with 1% Triton X-100 in PBS, rinsed three times with PBS, and blocked for 1 h in a blocking solution containing 2% (w/v) bovine serum albumin (BSA, Sigma-Aldrich, #05470-5G) and 5% (v/v) Normal Donkey Serum (#ab7475, Abcam). The primary antibody, AF594-conjugated anti-α-SMA (#ab202368, Abcam), was diluted 1:200 in blocking solution and incubated for one hour in the dark at room temperature. After three PBS washes, nuclei were counterstained with 2 μg mL^−1^ DAPI (#D9564-10MG, Sigma-Aldrich) for 5 min, followed by three final PBS washes and stored at 4 °C until imaging.

For EDA/EDB-FN visualization, samples previously stained with Cy5-labeled FnBPA5 and FN-AF488 were processed similarly, by leveraging the remaining open fluorescence channels. The protocol followed the same steps as the α-SMA staining with two modifications. First, the permeabilization step was omitted because EDA/EDB-FN is localized within the extracellular space. Second, the final DAPI counterstaining was excluded. The primary antibody used was specific to the EDA domain (clone IST-9, #sc-59826; Santa Cruz Biotechnology). Following incubation with the primary antibody and three PBS washes, secondary staining was performed in the same blocking solution using a goat anti-mouse AF555 secondary antibody (#A-21424, Invitrogen) at a 1:200 dilution for 1 h at RT in the dark. The secondary antibody was removed by three final washes with PBS before sample storage at 4 °C.

#### Confocal microscopy

When lung tissue overviews were not acquired with the Pannoramic 250 3DHISTECH slide scanner, stitched overview images were captured on a Nipkow spinning disk confocal microscope using a 10 × 0.45 NA CFI Plan Apo λ air objective, as shown in Fig. S5. Higher-resolution regions of interest were acquired either on a Leica TCS SP8 MP inverted multiphoton laser scanning microscope or on the Nipkow spinning disk confocal microscope using a 20 × 0.75 NA CFI Plan Apo λ air objective, as shown in Figs. S6Aiii and S11Bi. For the Leica SP8 system, imaging was conducted using either a 20 × 0.7 NA PH2 HC PLAN APO dry objective for Figs. 3Bi, 4A, and S7, or a 25 × 0.95 NA HCX IRAPO L water immersion objective for Figs. 1Cii, 2B, 3C, S3B, 4B, S8, and S12.

Fluorophores on the Leica SP8 system were excited in single-photon mode for FnBPA5-Cy5, FN-AF488, EDA/EDB-FN, and DAPI channels, whereas second-harmonic generation imaging was performed in two-photon mode. The samples were excited sequentially, except for FnBPA5-Cy5 and FN-AF488, which were excited simultaneously using two distinct laser lines, with signal collection divided between separate photomultiplier tubes. Mature collagen fibers were visualized exclusively with the 25× objective on the Leica SP8 system via second harmonic generation imaging in multiphoton laser scanning mode with an open pinhole. A Mai Tai XF from Spectra Physics femtosecond Ti-sapphire pulsed laser tuned to 880 nm was used to generate second-harmonic generation signals, which were detected at 440 nm.

Unless specified otherwise in the figure captions, the default acquisition setting across all microscopes and objectives was a z-stack of three slices with a 1 μm step size, maintaining a constant z-stack thickness of 2 μm. The imaging settings and laser intensities were kept constant within individual experiments containing internal controls to ensure valid quantitative comparisons between the treatment and control conditions. The specific number of regions of interest acquired per stain and sample is detailed in the respective figure captions.

### Image analysis

All confocal images of the ECM from the *in vitro* assays were analyzed using a combination of RStudio (version 2026.01.1 + 403) and FIJI ImageJ (version 1.54p). Each z-stack slice was individually imported into R, where a 3 × 3 low-pass averaging filter was applied to remove high-frequency noise. Pixel-by-pixel ratios were calculated by dividing the signal intensity of the peptide by that of FN in each slice, followed by averaging over all the slices of the stack. Color-coded heatmaps of the pixel-by-pixel intensity ratios were generated in FIJI using the look-up table “royal” scaled as indicated in the main and supplementary figures.

Ratiometric pixel heatmaps of mouse lung tissue cryosections were generated exclusively using FIJI. Ratios were calculated using the internal image calculator tool of FIJI in the “divide” mode. These ratio images were scaled as indicated in the figures using the same look-up table “royal” as for the *in vitro* images, which was performed for both the whole-tissue images from the slide scanner and the stitched overview images acquired with the low-resolution Nipkow 10× air objective.

### Statistical analysis

Specific statistical tests and significance levels are defined within the respective figure captions for all the main and supplementary panels.

## Statement on usage of generative AI

During the preparation of this work, the authors used Microsoft Copilot, Gemini, and Paperpal to improve the readability of the text. After using this tool/service, the authors reviewed and edited the content as needed and take full responsibility for the content of the publication.

## CRediT authorship contribution statement

**Konstantin M.P. Wolf:** Writing – review & editing, Writing – original draft, Visualization, Methodology, Investigation, Formal analysis, Conceptualization. **Emmanouil Angelidakis:** Writing – review & editing, Writing – original draft, Visualization, Methodology, Investigation, Formal analysis, Conceptualization. **Daria Künzli:** Writing – review & editing, Visualization, Methodology, Investigation, Formal analysis, Conceptualization. **David Lauer:** Writing – review & editing, Visualization, Methodology, Investigation, Formal analysis, Conceptualization. **Matthias Brunner:** Writing – review & editing, Methodology, Investigation, Conceptualization. **Alessia Cambria:** Writing – review & editing, Methodology, Investigation. **Divya Vats:** Writing – review & editing, Methodology, Investigation, Formal analysis, Conceptualization. **Britta Maurer:** Writing – review & editing, Methodology, Investigation, Conceptualization. **Viola Vogel:** Writing – review & editing, Writing – original draft, Supervision, Project administration, Funding acquisition, Conceptualization. **Mamta Chabria:** Writing – review & editing, Writing – original draft, Visualization, Supervision, Project administration, Methodology, Investigation, Funding acquisition, Formal analysis, Conceptualization.

## Declaration of competing interest

The authors declare the following financial interests/personal relationships which may be considered as potential competing interests: MC currently serves as a co-founder and CEO of TANDEM Therapeutics. MC declares a significant financial interest in this company, and this potential conflict of interest has been managed by proper disclosure and approval by ETH Zurich. VV serves as a co-founder of TANDEM Therapeutics and declares a financial interest in this company. BM declares research grants from AbbVie, Protagen, and Novartis Biomedical Research; declares a miR-29 patent for the treatment of systemic sclerosis (US8247389, EP2331143); declares lecturing fees from Boehringer Ingelheim, GlaxoSmithKline, Novartis, Otsuka, and Merck Sharpe & Dohme; declares consulting fees from Novartis, Boehringer Ingelheim, Janssen-Cilag, and GlaxoSmithKline; declares financial support for congresses (ie, for registration fees and travel) from Medtalk, Pfizer, Roche, Actelion, Mepha, and Merck Sharpe & Dohme; and is on the advisory board for Janssen and Boehringer.

## Data Availability

The raw and processed data supporting the findings of this study are available from the last author upon reasonable request, subject to confidentiality restrictions associated with the funding grant.

## Appendix A Supplementary data

Supplementary material Appendix A contains all supplementary figures (Figures S1-S12) referenced in this work.
