# Supplementary material for: Fibronectin's tensional state as a mechanical signature of fibrotic extracellular matrix in idiopathic pulmonary fibrosis models
Source: Matrix Biol Plus. 2026 Aug 25;32:100204. doi: 10.1016/j.mbplus.2026.100204 (PMC13563565; doi:10.1016/j.mbplus.2026.100204)
Supplement: Supplementary file 1 [file mmc1.pdf]

# Supplementary Information

## Fibronectin's Tensional State as a Mechanical Signature of Fibrotic Extracellular Matrix in Idiopathic Pulmonary Fibrosis Models

Konstantin M. P. Wolf[1,4], Emmanouil Angelidakis[1,4], Daria Künzli[1,4], David Lauer[2,3], Matthias Brunner[2,3], Alessia Cambria[1], Divya Vats[1], Britta Maurer[2,3], Viola Vogel[1] & Mamta Chabria[1,*]

[1]Laboratory of Applied Mechanobiology, Institute of Translational Medicine, Department of Health Sciences and Technology, ETH Zurich, Switzerland

[2]Department of Rheumatology and Immunology, Inselspital, Bern University Hospital, University of Bern, Bern Switzerland

[3]Lung Precision Medicine (LPM), Department of BioMedical Research (DBMR), University of Bern, Bern, Switzerland

[4]These authors contributed equally

*corresponding author

**Contact:**

Dr. Mamta Chabria (mamta.chabria@wysszurich.ch), ETH Zürich, Tandem Therapeutics, Wyss Zürich Translational Center, Südstrasse 1, d92, 8952, Schlieren, Switzerland

**Keywords:** extracellular matrix, fibronectin, mechanics, idiopathic pulmonary fibrosis

**This file includes:**

Supplementary Figures

Captions for Supplementary Figures

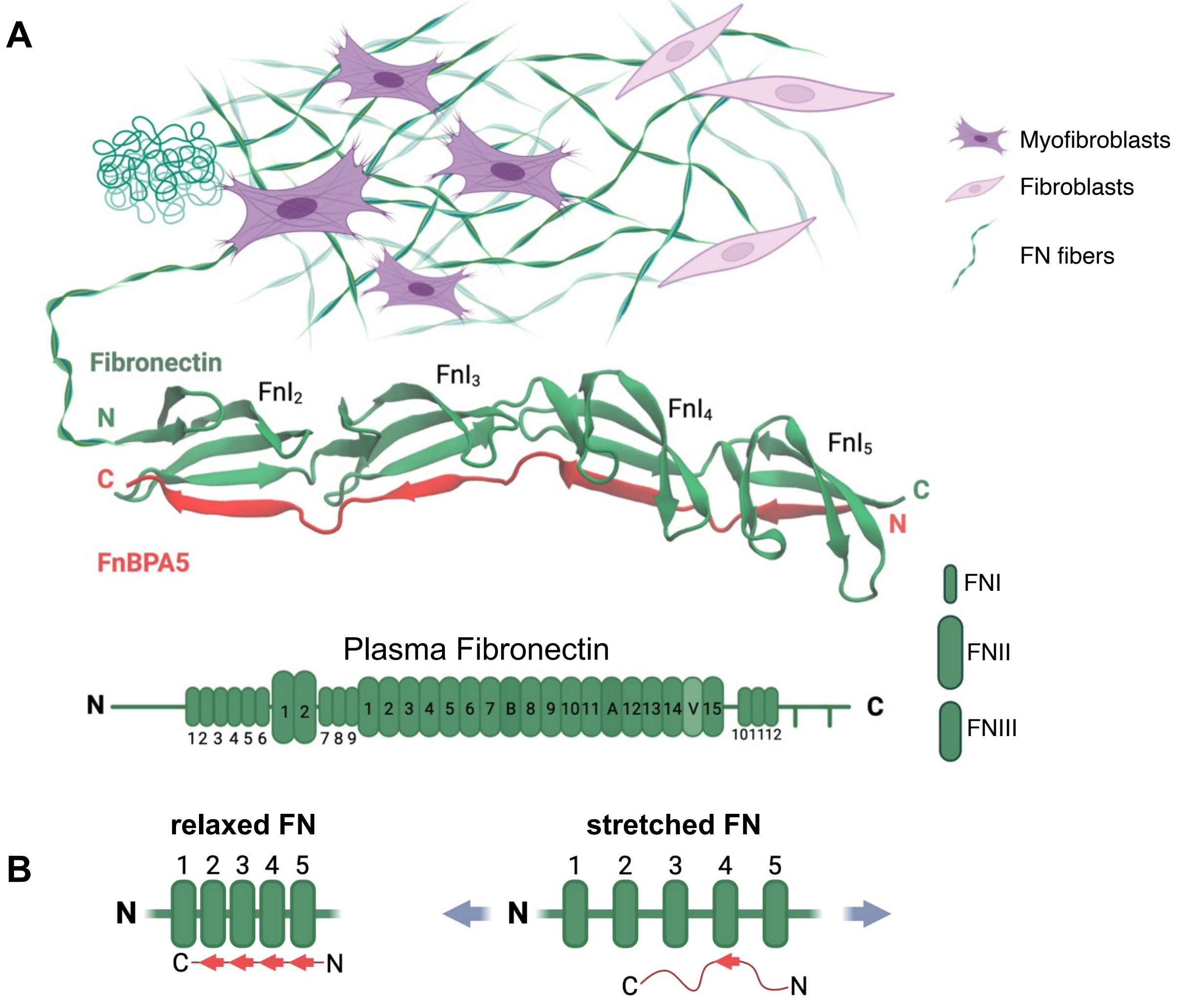


**Figure S1 | Tension-sensitive binding mode of FnBPA5 to fibronectin's N-terminal type I modules.**

Fibronectin is a major fibrous ECM protein with modular structure assembled by fibroblasts and more efficiently by myofibroblasts (**A**). The bacterially derived FnBPA5 peptide forms a multivalent complex with the N-terminal fibronectin type $I_{2\text{-}5}$ modules which is destroyed by stretching the fibronectin fiber, as structurally predicted by steered molecular dynamics simulations and validated in stretch assays[1,2] and later in cell culture[3] (**B**).

Figure adapted from Arnoldini *et al.*[3].

***References:***

[1]*Chabria, M., Hertig, S., Smith, M. L. & Vogel, V. Stretching fibronectin fibers disrupts binding of bacterial adhesins by physically destroying an epitope. Nat Commun 1, 135, doi:10.1038/ncomms1135 (2010).*

[2]*Hertig, S., Chabria, M. & Vogel, V. Engineering mechanosensitive multivalent receptor-ligand interactions: why the nanolinker regions of bacterial adhesins matter. Nano Lett 12, 5162-5168, doi:10.1021/nl302153h (2012).*

[3]*Arnoldini, S., Moscaroli, A., Chabria, M., Hilbert, M., Hertig, S., Schibli, R., Béhé, M. & Vogel, V. Novel peptide probes to assess the tensional state of fibronectin fibers in cancer. Nat Commun 8, 1793, doi:10.1038/s41467-017-01846-0 (2017).*

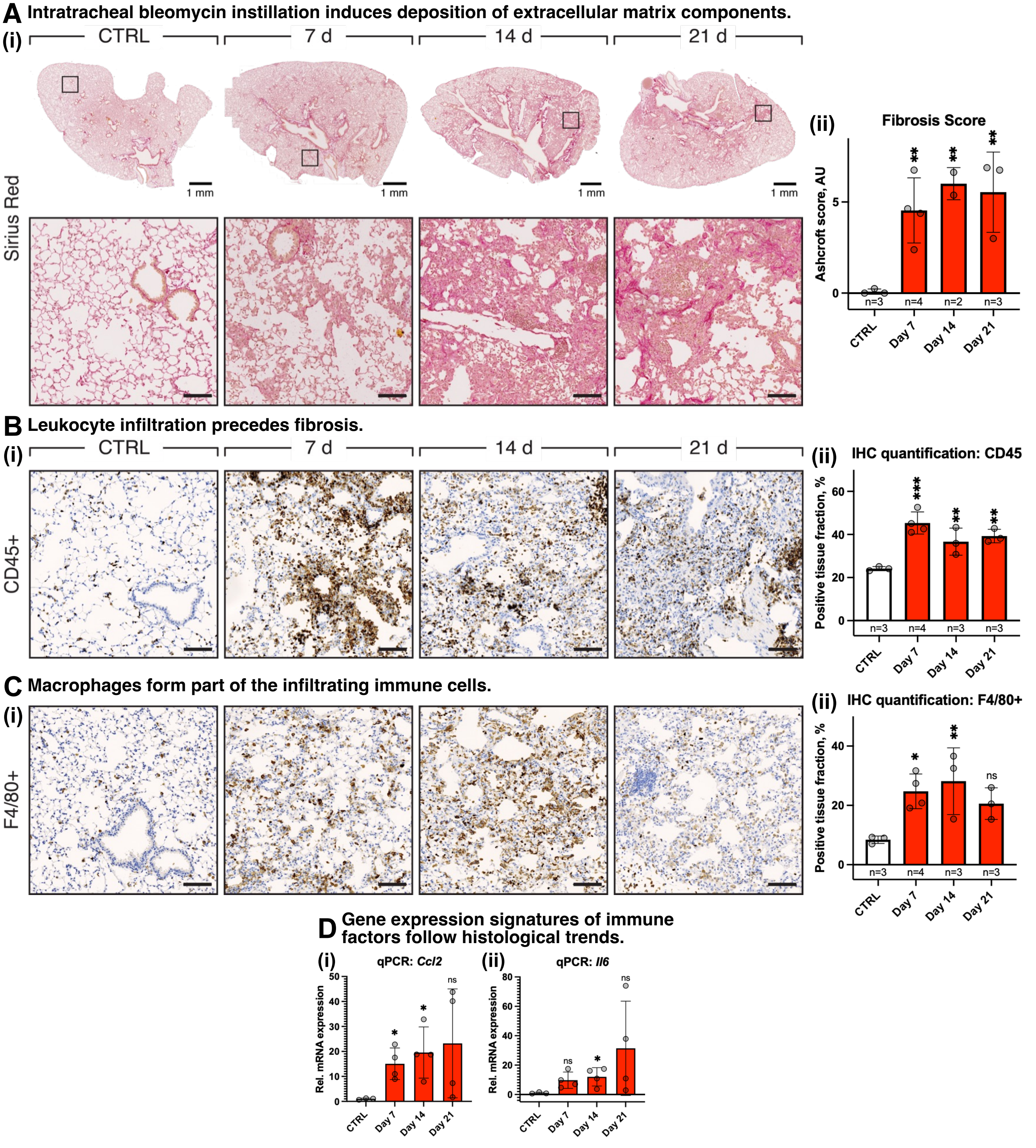


**Figure S2 | Time-resolved development of inflammation and lung fibrosis in the bleomycin-induced mouse model.**

**(A) Fibrosis progression.** (i) Picrosirius Red staining of lung tissue sections (right inferior lobe) from representative mice across four conditions: CTRL (21 days intratracheal PBS) and 7, 14, and 21 days after intratracheal bleomycin instillation. Insets show selected 500 × 500 $\mu$m regions. Scale bars: 1 mm (overview), 100 $\mu$m (magnified). (ii) Semiquantitative Ashcroft scores of Picrosirius Red-stained right inferior lobes for all mice at each time point.

**(B) Leukocyte infiltration.** (i) Representative immunohistochemistry images stained for the pan-leukocyte marker CD45 (right inferior lobe; 500 × 500 $\mu$m fields; scale bar: 100 $\mu$m). (ii) Quantification of $CD45^+$ cell infiltration for all mice per treatment group.

**(C) Macrophage infiltration.** (i) Representative immunohistochemistry images stained for the murine macrophage marker F4/80 (right inferior lobe; 500 × 500 $\mu$m fields; scale bar: 100 $\mu$m). (ii) Quantification of F4/80$^+$ macrophages for all mice per treatment group.

Across panels A(ii), B(ii), and C(ii), each data point represents an individual mouse; group sizes (n) are indicated below each bar plot.

**(D) Time-resolved gene expression.** RT-qPCR quantification of immune-related genes (*Ccl2*, *Il6*) from right superior lobes. Expression was normalized to the housekeeping gene *Rplp0*. RT-qPCRs were performed on three biological replicates (three mice) for CTRL and four biological replicates (four mice) for days 7, 14, and 21 post-bleomycin.

**Statistics.** Normality was assessed by QQ-plot (Aii) or Shapiro-Wilk test (Bii, Cii, D). Variance homogeneity was tested using Levene's test. For normally distributed data with homogeneous variance, significance was assessed using unpaired two-tailed multiple-comparison t-tests (Aii, Bii, Cii). For data with non-homogeneous variance, significance was assessed via Welch's ANOVA and Welch's t-test (D). P-values < 0.05 were considered significant (* $p < 0.05$; ** $p < 0.01$; *** $p < 0.001$; **** $p < 0.0001$).

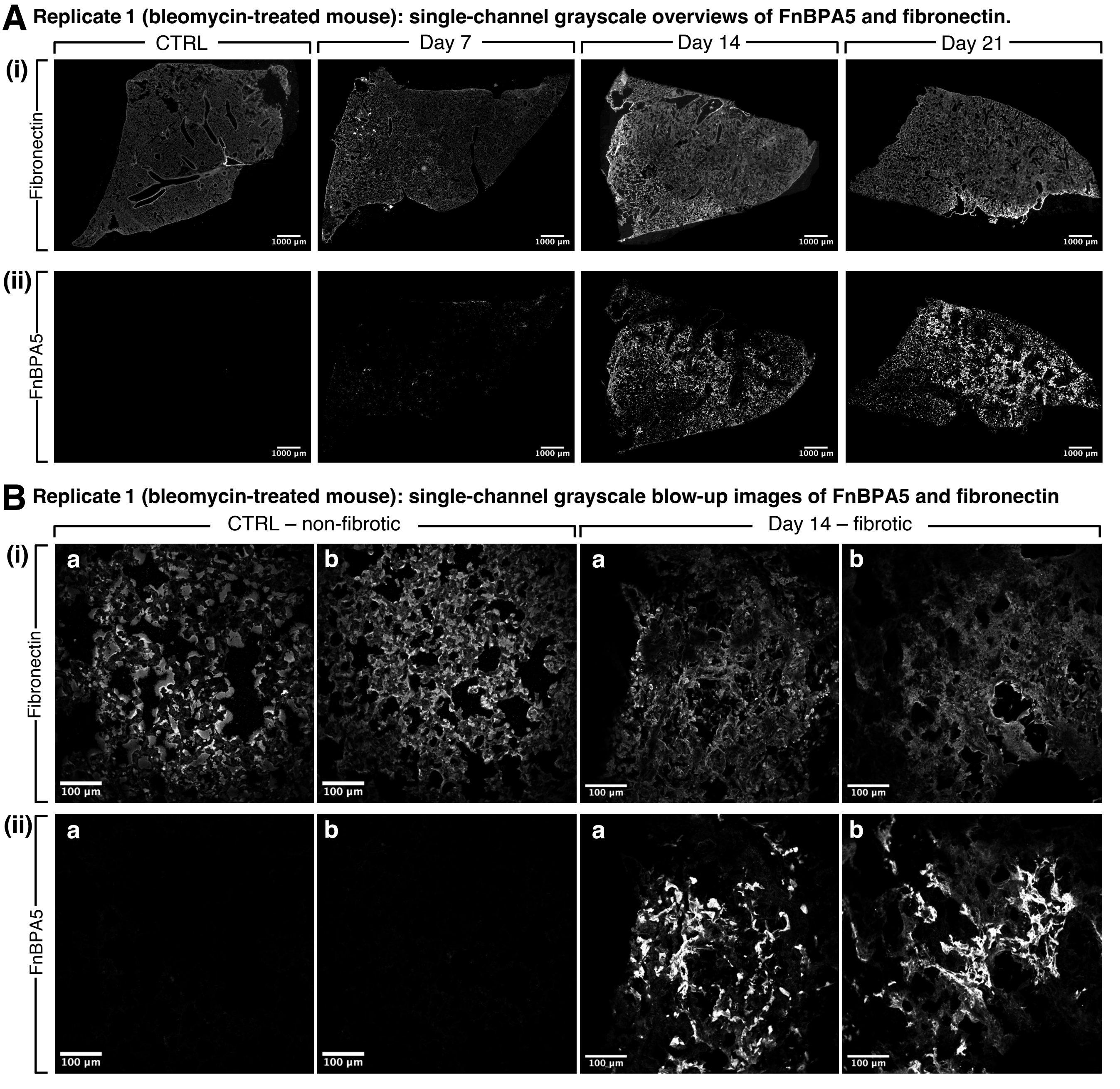


**Figure S3 l Single channel grayscale images of fibronectin and FnBPA5 staining (replicate 1 corresponding to Figure 1)**

Single channel grayscale images corresponding to the composite images in Figure 1 are shown to allow separate visual assessment of the fibronectin and FnBPA5 signal contributions.

(A) Whole tissue overview images for all experimental time points (CTRL, day 7, day 14, and day 21 post-bleomycin instillation) showing (i) the fibronectin channel and (ii) the FnBPA5 channel; these correspond to Figure 1Ci.

(B) Single channel images of the magnified regions of interest (a and b) indicated in Figure 1Ci, shown for CTRL and day 14, corresponding to Figure 1Cii.

Overview images in (A) were acquired on a 3DHISTECH Pannoramic 250 slide scanner using a 20× air Plan Apochromat objective. Magnified images in (B) were acquired on a Leica SP8 line scanning confocal microscope using a 25× 0.95 NA HCX IRAPO L water immersion objective.

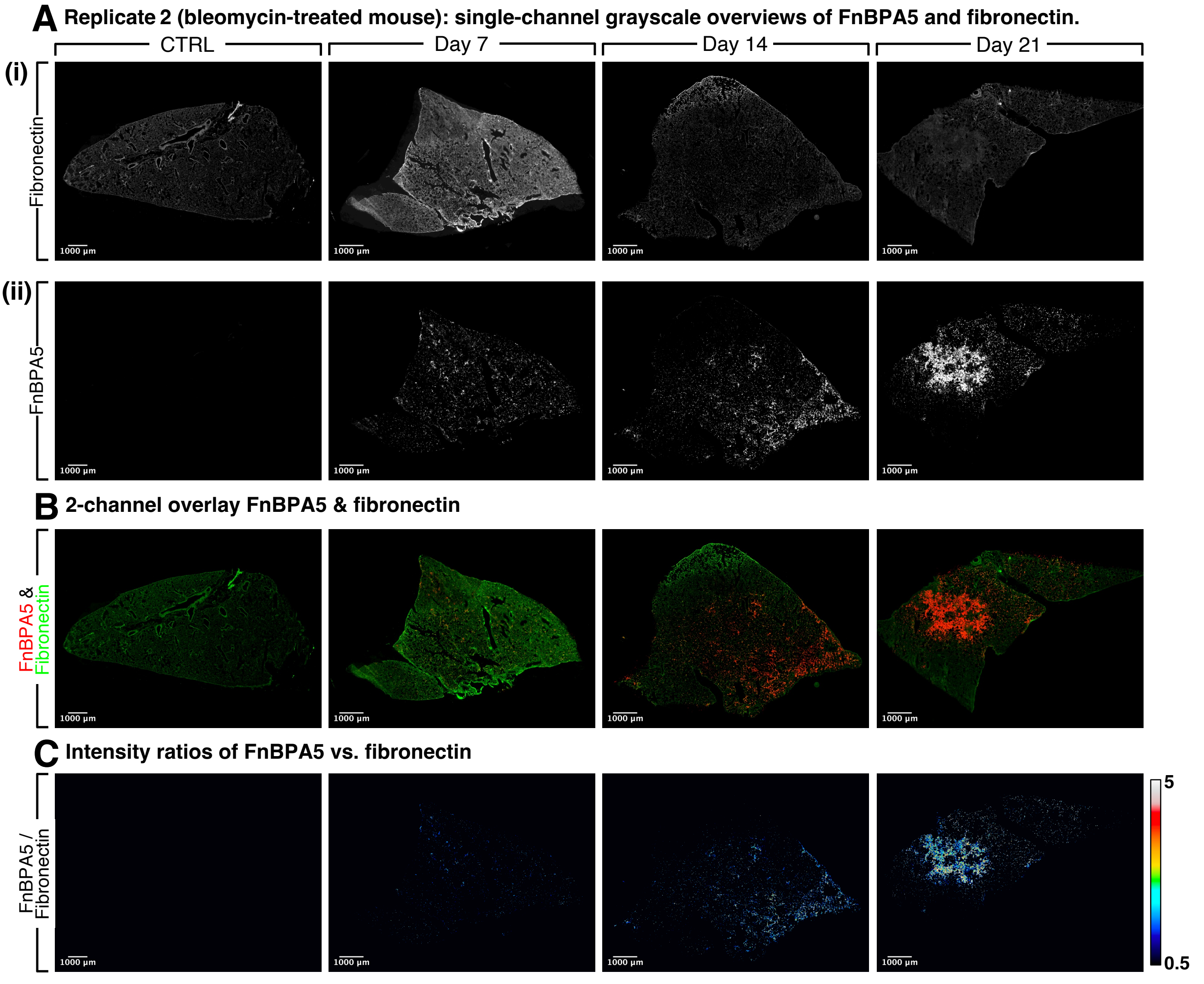


**Figure S4 I Second mouse replicate for FnBPA5-Cy5 and fibronectin staining across all time points.**

This figure presents the second biological replicate for each experimental time point stained for FnBPA5-Cy5 and fibronectin, using the same staining protocol as in Figure 1C.

(A) Single-channel grayscale images showing (i) the fibronectin signal and (ii) the FnBPA5 signal, displayed to enable separate assessment of individual channel contributions, corresponding to the presentation of replicate 1 in Figure S3.

(B) Two-color overlay images generated from the channels shown in (A).

(C) Ratio images calculated from the single-channel data using the FIJI Image Calculator function "Divide" (FnBPA5 channel divided by fibronectin channel), visualized using the "royal" lookup table as indicated.

All tissues were imaged on a 3DHISTECH Pannoramic 250 slide scanner using a 20× air Plan Apochromat objective, as in Figure 1Ci and S3. The staining procedure, imaging settings, and FIJI processing steps were carried out identically for all eight tissue samples, ensuring direct comparability across time points and between biological replicates.

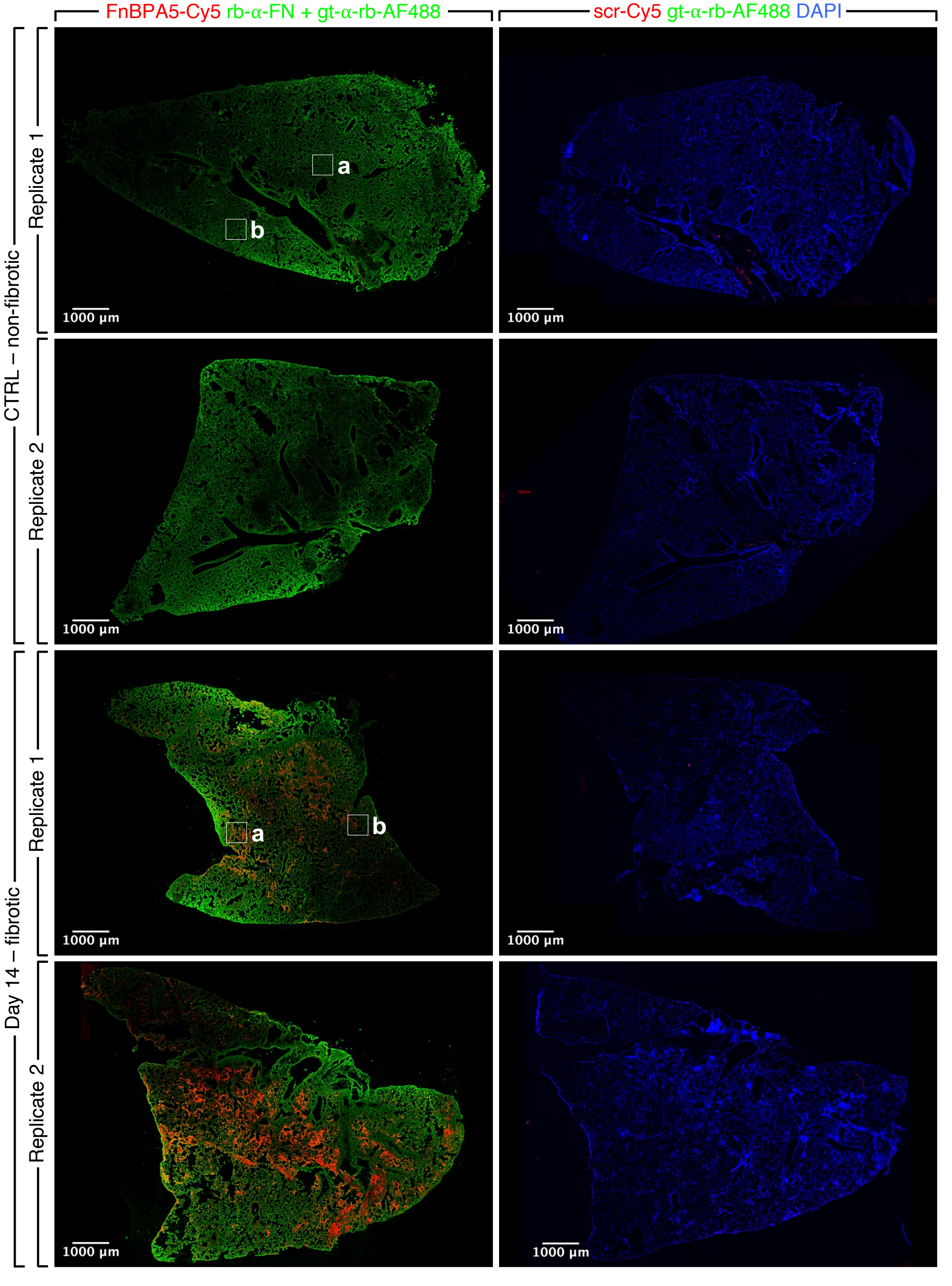


**Figure S5 | Background controls for FnBPA5-Cy5 and fibronectin staining (scrambled peptide; primary-antibody omission)**

Background controls corresponding to the samples shown in Figures 1Ci and S4 are presented. Tissue allocation was as follows: for CTRL replicate 1, cryosections adjacent to Figure S4 (CTRL) were used; for CTRL replicate 2, cryosections adjacent to Figure 1Ci (CTRL) were used; for day 14 post-bleomycin instillation replicate 1, a section from a new mouse was used; and for day 14 post-bleomycin instillation replicate 2, a section adjacent to Figure 1Ci (day 14) was used.

To compare full staining with background, parallel stainings were performed on adjacent tissue sections from each replicate: **Full staining (left column)**: FnBPA5-Cy5 combined with fibronectin primary antibody and AF488-labeled secondary antibody, carried out in two biological replicates. **Background controls (right column)**: Identical staining protocol with the same reagent volumes but replacing FnBPA5-Cy5 with a Cy5-labeled scrambled peptide (scr-Cy5) of identical amino-acid composition and omitting the primary fibronectin antibody for the AF488 channel. These controls report tissue autofluorescence and non-specific secondary antibody binding, providing a direct reference to confirm that the observed FnBPA5 and fibronectin signals are genuine.

All samples (full stainings and background controls) were processed and imaged in parallel on the same microscope, using identical imaging settings (laser power, exposure time) as well as identical FIJI processing steps and image display settings, ensuring direct comparability. Control samples included DAPI to visualize tissue structure, as the background from scr-Cy5 and secondary antibody alone was minimal. DAPI was not included in the full staining shown in the left column. Images show stitched tissue overviews acquired with a 10× air objective (0.45 CFI Plan Apo λ) on a Nipkow spinning disk confocal microscope. The abbreviations “rb” and “gt” refer to the host species of the antibodies, where rb = rabbit (primary antibody) and gt = goat (AF488-labeled secondary antibody).

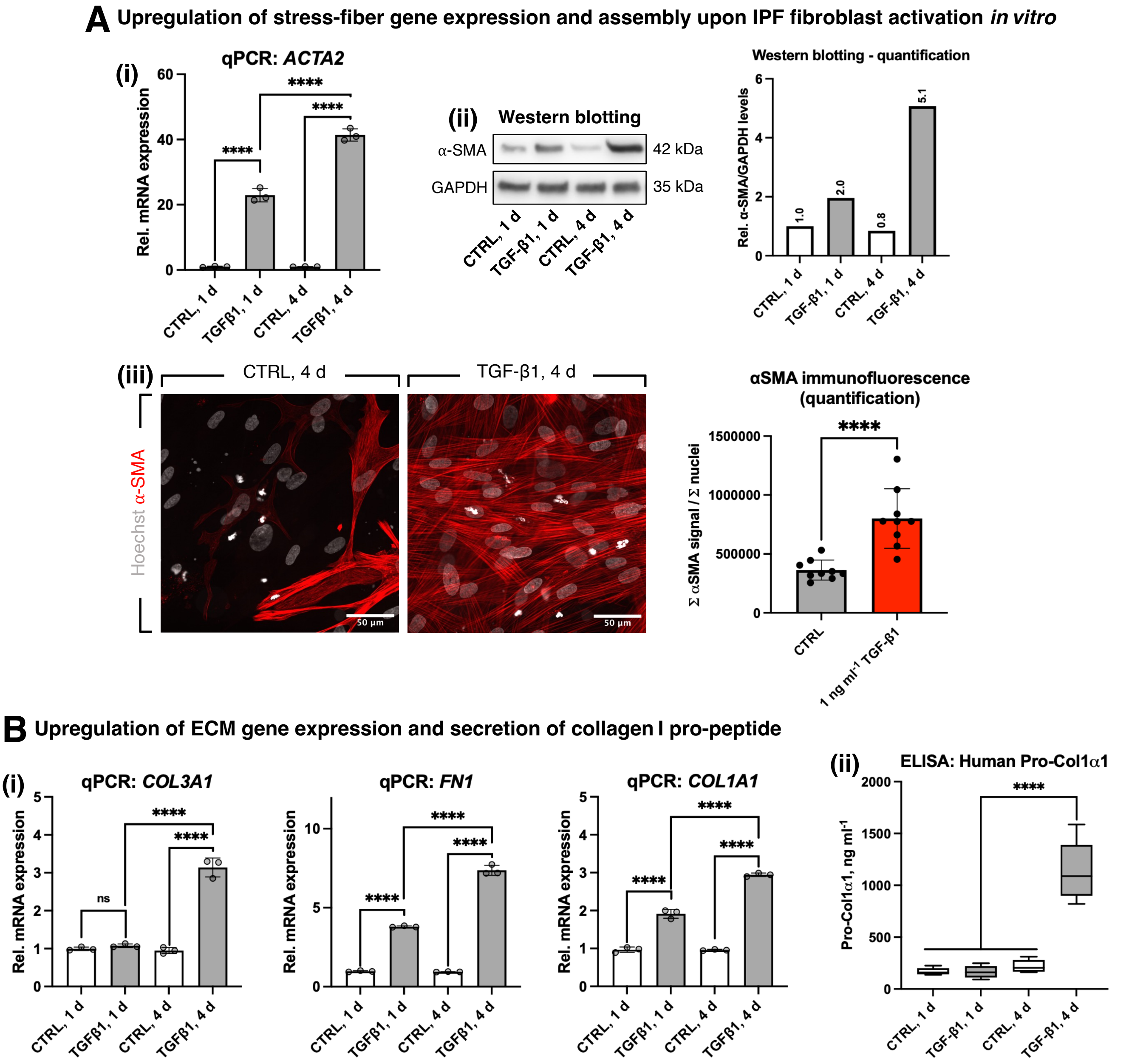


**Figure S6 I Quantification of fibrotic markers associated with the fibroblast-to-myofibroblast transition *in vitro*.**

Analyses were performed on samples collected following the same stimulation timeline as in Figure 3A (day 1 and day 4, ± 1 ng ml$^{-1}$ TGF-β1).

**(A) Upregulation of stress-fiber gene expression and assembly upon fibroblast activation *in vitro*.**

(i) *ACTA2* (encoding α-smooth muscle actin, α-SMA) expression quantified by RT-qPCR and normalized to *RPLP0*.

(ii) α-SMA protein levels assessed by Western Blot of whole-cell lysates (loading control: GAPDH). Data represent two independent experiments. Protein band intensities were quantified densitometrically in FIJI, and α-SMA/GAPDH ratios are displayed as bar plots.

(iii) α-SMA stress fibers were visualized by immunofluorescence (α-SMA in red; nuclei in grey) using a Nipkow spinning-disk confocal microscope equipped with a 20× air objective. α-SMA signal intensities were quantified with a FIJI macro that sums the total α-SMA fluorescence per image and normalizes it to the number of nuclei detected by StarDist 2D (default settings; pixel-area cutoff of 35). IPF fibroblasts from donor 1 were seeded in three wells per condition, and three regions of interest were imaged per well. Plotted values represent total α-SMA signal per image normalized to the number of detected nuclei.

**(B) Upregulation of ECM gene expression and secretion of collagen I pro-peptide upon fibroblast activation *in vitro*.**

(i) Expression of *COL3A1* (collagen III), *FN1* (fibronectin), and *COL1A1* (collagen I) quantified by RT-qPCR and normalized to *RPLP0*.

(ii) N-terminal collagen I α1 pro-peptide levels in culture supernatants measured by ELISA. ELISA data represent two independent experiments measured in two wells each, with technical duplicates (n = 8).

RT-qPCR data in (A) and (B) represent two independent experiments, each performed in technical triplicates.

**Statistics.**

(Ai, B) Normality was assessed using the Shapiro–Wilk test and equality of variance using Levene's test. For normally distributed data with equal variance, significance was determined using unpaired, two-sided multiple-comparison t-tests with Benjamini–Hochberg correction.

(Aiii) Non-parametric comparisons were performed using the Mann–Whitney test.

A p-value < 0.05 was considered significant (*p < 0.05; **p < 0.01; ***p < 0.001; ***p < 0.0001).

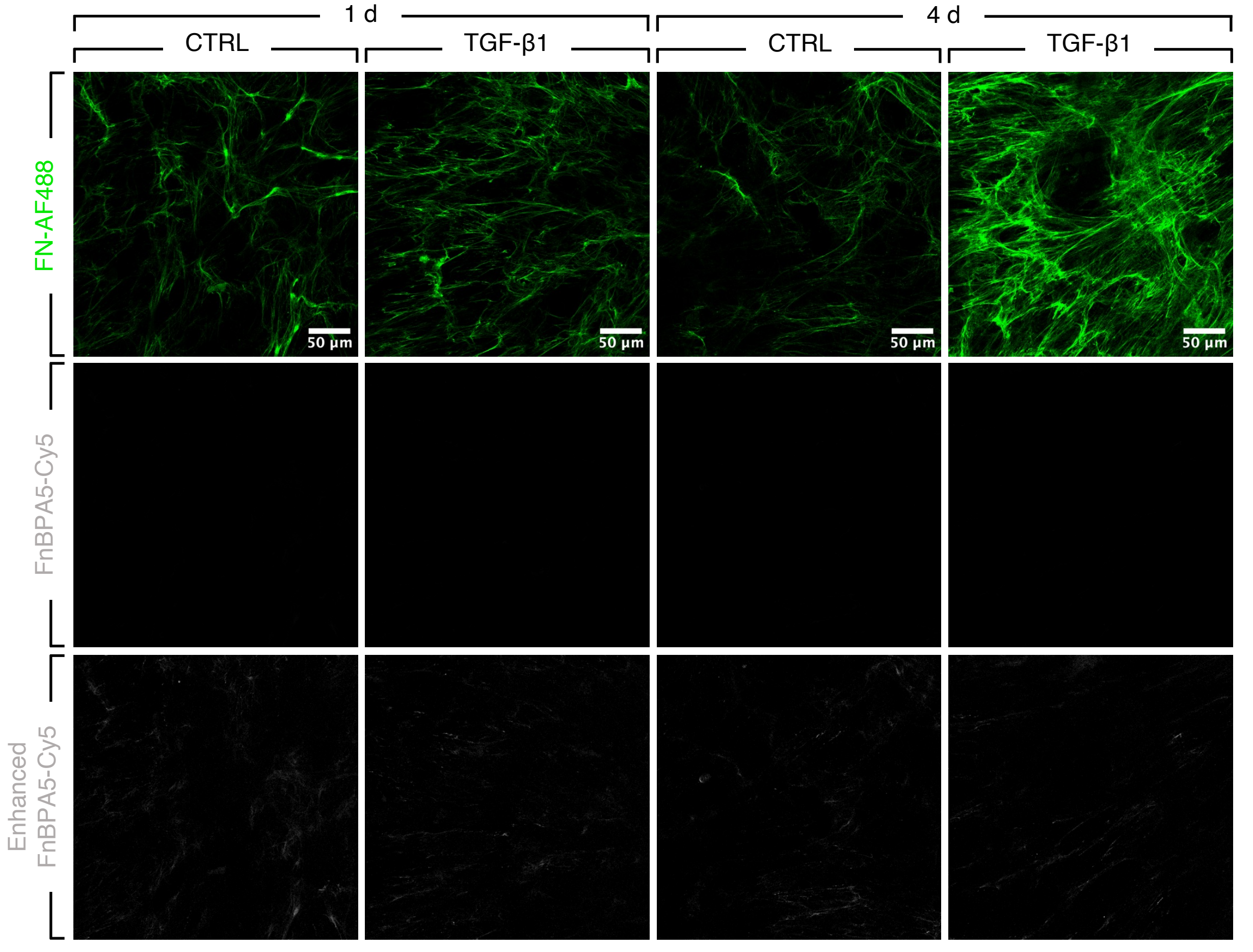


**Figure S7 | Non-specific binding of scr-Cy5 to fibronectin in ECM derived from IPF patient-derived human lung fibroblasts.**
A scrambled control peptide with the same amino-acid composition as FnBPA5 was labelled with Cy5 (scr-Cy5) to assess non-specific peptide binding to fibronectin. The fibronectin fibrillar network was visualized by supplementing cultures with FN-AF488, which is incorporated into the extracellular matrix during *de novo* ECM assembly. After ECM formation, samples were incubated with scr-Cy5, fixed, and imaged. Shown are representative confocal images (acquired and processed using the same settings as in Figure 3) of fibronectin and scrambled-peptide staining for cells cultured with or without TGF-β1 at day 1 and day 4. Additionally, scr-Cy5 images with strongly enhanced contrast are provided to highlight low-level background signals.

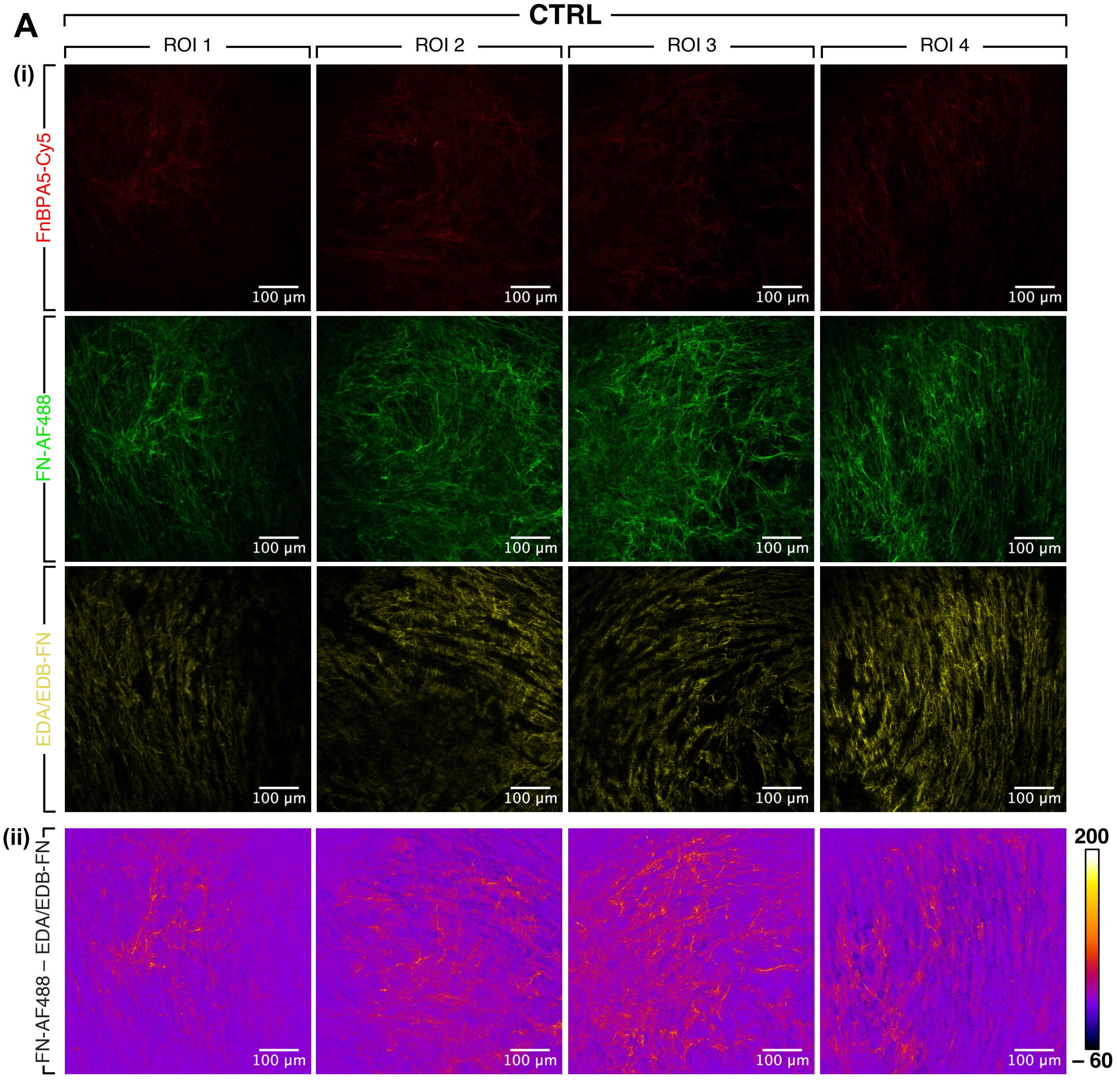
A
CTRL
ROI 1
ROI 2
ROI 3
ROI 4
(i)
FnBPA5-Cy5
FN-AF488
EDA/EDB-FN
(ii)
FN-AF488 – EDA/EDB-FN
100 µm
200
– 60

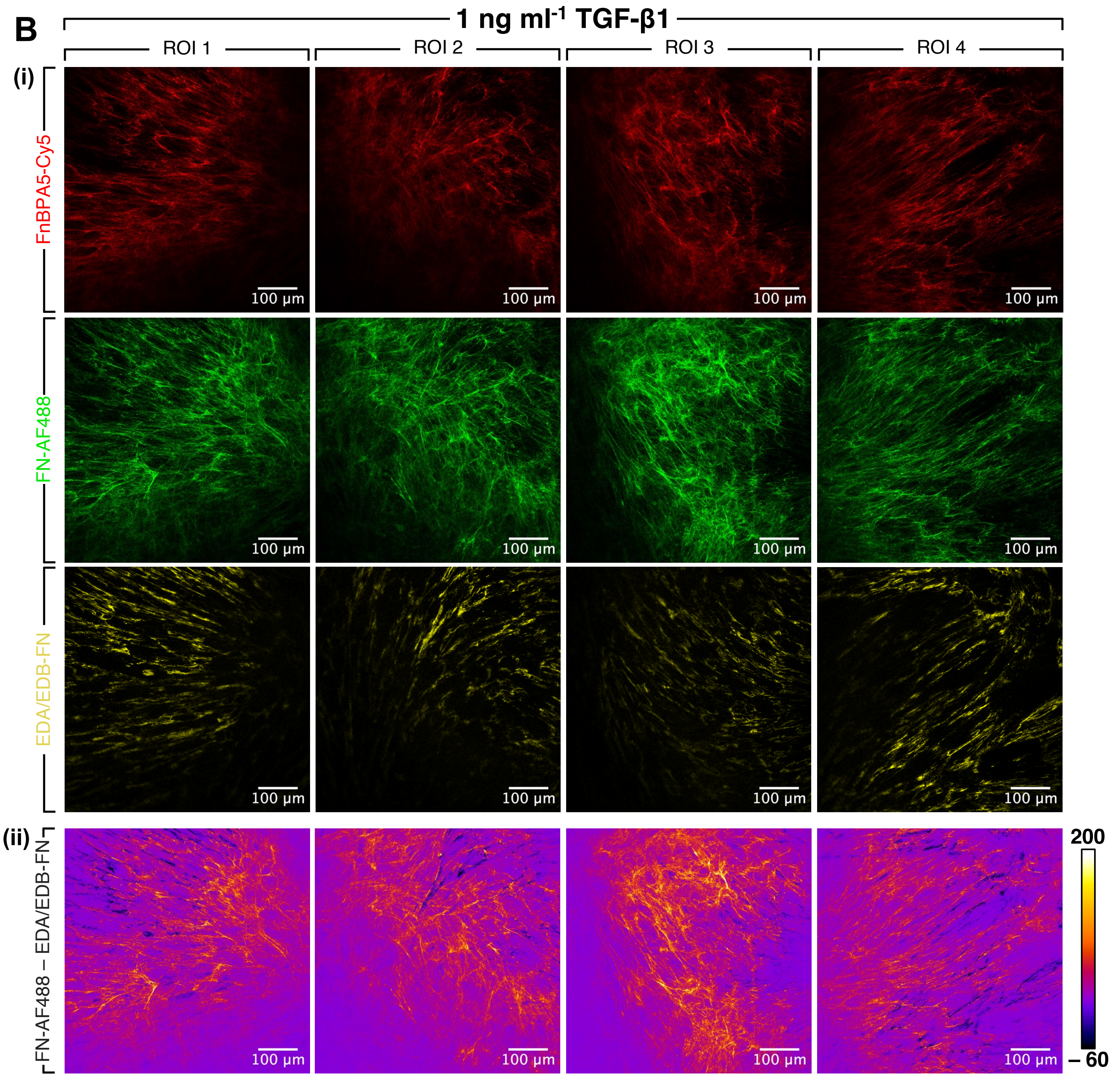

B
1 ng ml-1 TGF-β1
ROI 1
ROI 2
ROI 3
ROI 4
(i)
FnBPA5-Cy5
FN-AF488
EDA/EDB-FN
(ii)
FN-AF488 – EDA/EDB-FN
100 µm
200
– 60

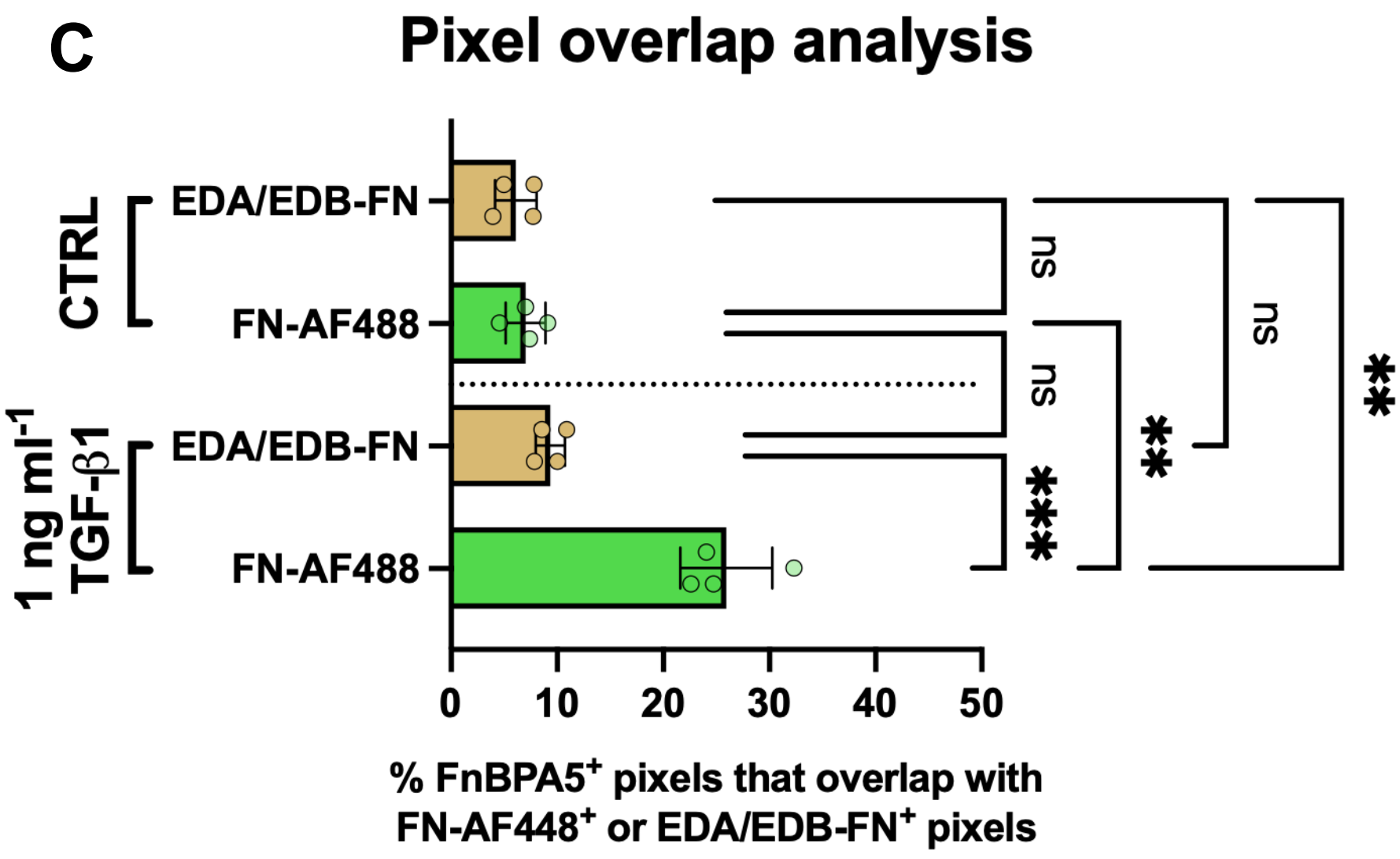


**Figure S8 | Assembly of cell-derived EDA/EDB-containing FN fibers is upregulated upon TGF-β1, while relaxed FN fibers reported by FnBPA5-Cy5 are primarily plasma-derived.**

IPF cells (donor 2) were cultured with FN-AF488 and treated according to the timeline in Figure 3A. Following FnBPA5-Cy5 incubation and fixation, cellular FN fibers were immunolabeled using antibodies specific to EDA/EDB-FN isoforms.

(A, B) Representative confocal images of cells treated (A) without (CTRL) or (B) with 1 ng $ml^{-1}$ TGF-β1. Z-stack images (5 planes) were acquired on a Leica SP8 line-scanning confocal microscope (25×, 0.95 NA HCX IRAPO L water-immersion objective). Laser intensities and gains were individually optimized for each channel to utilize the full 8-bit dynamic range (0–255) with minimal clipping to allow for post-processing steps as described below. Images display the central slice (plane 3), selected for maximal signal intensity along the z-axis. While the FnBPA5-Cy5 signal distribution closely mirrors FN-AF488, distinct differences occur between FN-AF488 and cellular EDA/EDB-FN. (ii) To highlight and quantify these local compositional differences, the EDA/EDB-FN signal was subtracted from the FN-AF488 signal at pixel resolution and visualized as "Fire" LUT heatmaps (FIJI; scale: -60 to 200). Dark structures represent predominantly cellular fibers; red-orange-yellow structures represent predominantly plasma-derived FN-AF488. Four independent regions of interest (ROIs) were imaged per condition.

(C) Quantitative analysis pooling all four ROIs per condition to evaluate how $FnBPA5^{+}$ pixels overlap with either plasma FN (FN-AF488) or cellular FN (EDA/EDB-FN). An R script converted images to matrices to determine the ratio of overlapping positive pixels between channel pairs. Background cutoffs were determined visually using the FIJI thresholding tool and applied in the R script (signal >20 for FnBPA5 and FN-AF488; signal >6 for EDA/EDB-FN). Overlap scores were plotted using Prism. Statistical significance was evaluated via a one-way ANOVA on log-transformed means with Tukey's multiple comparison test (* $p < 0.05$; ** $p < 0.01$; *** $p < 0.001$).

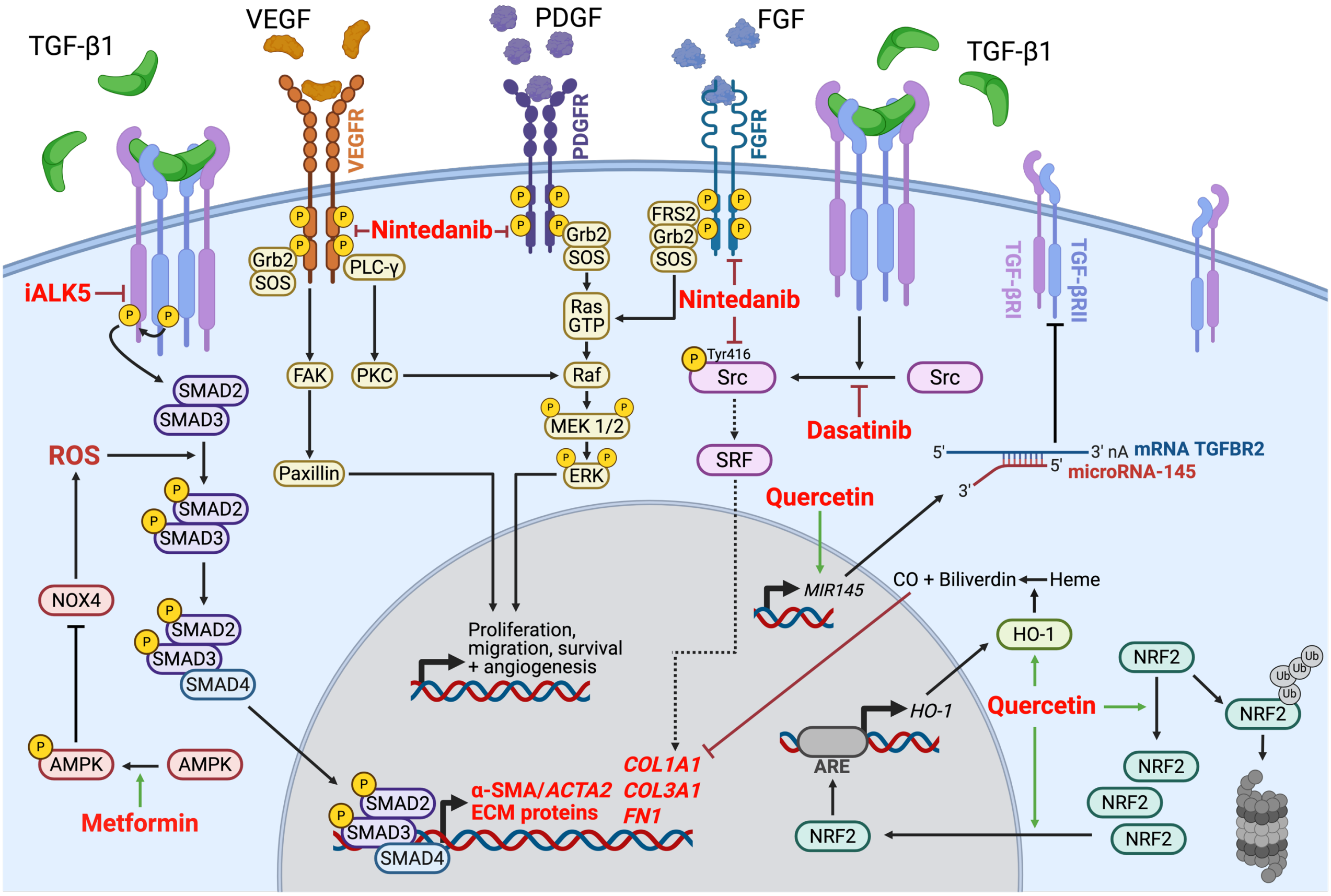


**Figure S9 I Targeting druggable vulnerabilities in signaling of idiopathic pulmonary fibrosis using drugs with reported antifibrotic potential.**

Accumulation of profibrotic myofibroblasts is a key process in IPF development and canonic TGF-β1 plays a major role in their emergence. Canonic TGF-β1 signaling is initiated upon TGF-β1 binding to the extracellular domain of TGF-βRII and dimerization with TGF-βRI. TGF-βRII phosphorylates TGF-βRI at its cytosolic domain, which mediates phosphorylation of downstream receptor-associated SMADs (R-SMADs), SMAD2 and SMAD3, which, after dimerization, associate with SMAD4 for expression of pro-fibrotic, myofibroblastic genes such as α-SMA/*ACTA2*, collagen 1/*COL1A1*, collagen 3/*COL3A1* and fibronectin/*FN1*. Non-canonic fibroblast activation in IPF comprises, amongst other pathways, VEGF, PDGF and FGF-signaling, as well as Src-SRF and redox signaling (NOX4, NRF2), which can further crosstalk with canonic TGF-β1 signaling. Figure is based on data from Grygielko *et al.* (iALK5)[1], Sato *et al.* and Kheirollahi *et al.* (metformin)[2,3], Nakamura *et al.* and Xu *et al.* (quercetin)[4,5], Wollin *et al.* (nintedanib)[6] and Abdalla *et al.* (dasatinib)[7] and was created with Biorender.com.

*Abbreviations: TGF-β receptor type 1 & 2: TGF-βRI & TGF-βRII; Small Mothers Against Decapentaplegic 2, 3, & 4: SMAD2, -3 & -4; Platelet-derived, fibroblast & vascular endothelial growth factor receptors: PDGFR, FGFR & VEGFR; Sarcoma kinase: Src; Serum reponse factor: SRF, Heme oxygenase 1: HO-1, Nuclear Factor Erythroid 2-related Factor 2: NRF2; AMP-activated protein kinase: AMPK; NADPH oxidase 4: NOX4; Growth factor receptor-bound protein 2: Grb2; Son of Sevenless: SOS; Focal adhesion kinase: FAK; Phospholipase-C, γ: PLC-γ, Fibroblast Growth Factor Receptor Substrate 2: FRS2, Rat sarcoma virus protein: Ras; Rat fibrosarcoma protein: Raf; Mitogen-activated protein kinase 1 & 2: MEK1/2; Extracellular-signal regulated kinases: ERK.*

***References:***


1 *Grygielko, E. T., Martin, W. M., Tweed, C., Thornton, P., Harling, J., Brooks, D. P. & Laping, N. J. Inhibition of gene markers of fibrosis with a novel inhibitor of transforming growth factor-beta type I receptor kinase in puromycin-induced nephritis. J Pharmacol Exp Ther 313, 943-951, doi:10.1124/jpet.104.082099 (2005).*

2 *Sato, N., Takasaka, N., Yoshida, M., Tsubouchi, K., Minagawa, S., Araya, J., Saito, N., Fujita, Y., Kurita, Y., Kobayashi, K., Ito, S., Hara, H., Kadota, T., Yanagisawa, H., Hashimoto, M., Utsumi, H., Wakui, H., Kojima, J., Numata, T., Kaneko, Y., Odaka, M., Morikawa, T., Nakayama, K., Kohrogi, H. & Kuwano, K. Metformin attenuates lung fibrosis development via NOX4 suppression. Respir Res 17, 107, doi:10.1186/s12931-016-0420-x (2016).*

3 *Kheirollahi, V., Wasnick, R. M., Biasin, V., Vazquez-Armendariz, A. I., Chu, X., Moiseenko, A., Weiss, A., Wilhelm, J., Zhang, J. S., Kwapiszewska, G., Herold, S., Schermuly, R. T., Mari, B., Li, X., Seeger, W., Günther, A., Bellusci, S. & El Agha, E. Metformin induces lipogenic differentiation in myofibroblasts to reverse lung fibrosis. Nat Commun 10, 2987, doi:10.1038/s41467-019-10839-0 (2019).*

4 *Nakamura, T., Matsushima, M., Hayashi, Y., Shibasaki, M., Imaizumi, K., Hashimoto, N., Shimokata, K., Hasegawa, Y. & Kawabe, T. Attenuation of transforming growth factor-β-stimulated collagen production in fibroblasts by quercetin-induced heme oxygenase-1. Am J Respir Cell Mol Biol 44, 614-620, doi:10.1165/rcmb.2010-0338OC (2011).*

5 *Xu, J., Tan, Y. L., Liu, Q. Y., Huang, Z. C., Qiao, Z. H., Li, T., Hu, Z. Q. & Lei, L. Quercetin regulates fibrogenic responses of endometrial stromal cell by upregulating miR-145 and inhibiting the TGF-β1/Smad2/Smad3 pathway. Acta Histochem 122, 151600, doi:10.1016/j.acthis.2020.151600 (2020).*

6 *Wollin, L., Wex, E., Pautsch, A., Schnapp, G., Hostettler, K. E., Stowasser, S. & Kolb, M. Mode of action of nintedanib in the treatment of idiopathic pulmonary fibrosis. Eur Respir J 45, 1434-1445, doi:10.1183/09031936.00174914 (2015).*

7 *Abdalla, M., Thompson, L., Gurley, E., Burke, S., Ujjin, J., Newsome, R. & Somanath, P. R. Dasatinib inhibits TGFβ-induced myofibroblast differentiation through Src-SRF Pathway. Eur J Pharmacol 769, 134-142, doi:10.1016/j.ejphar.2015.11.008 (2015).*

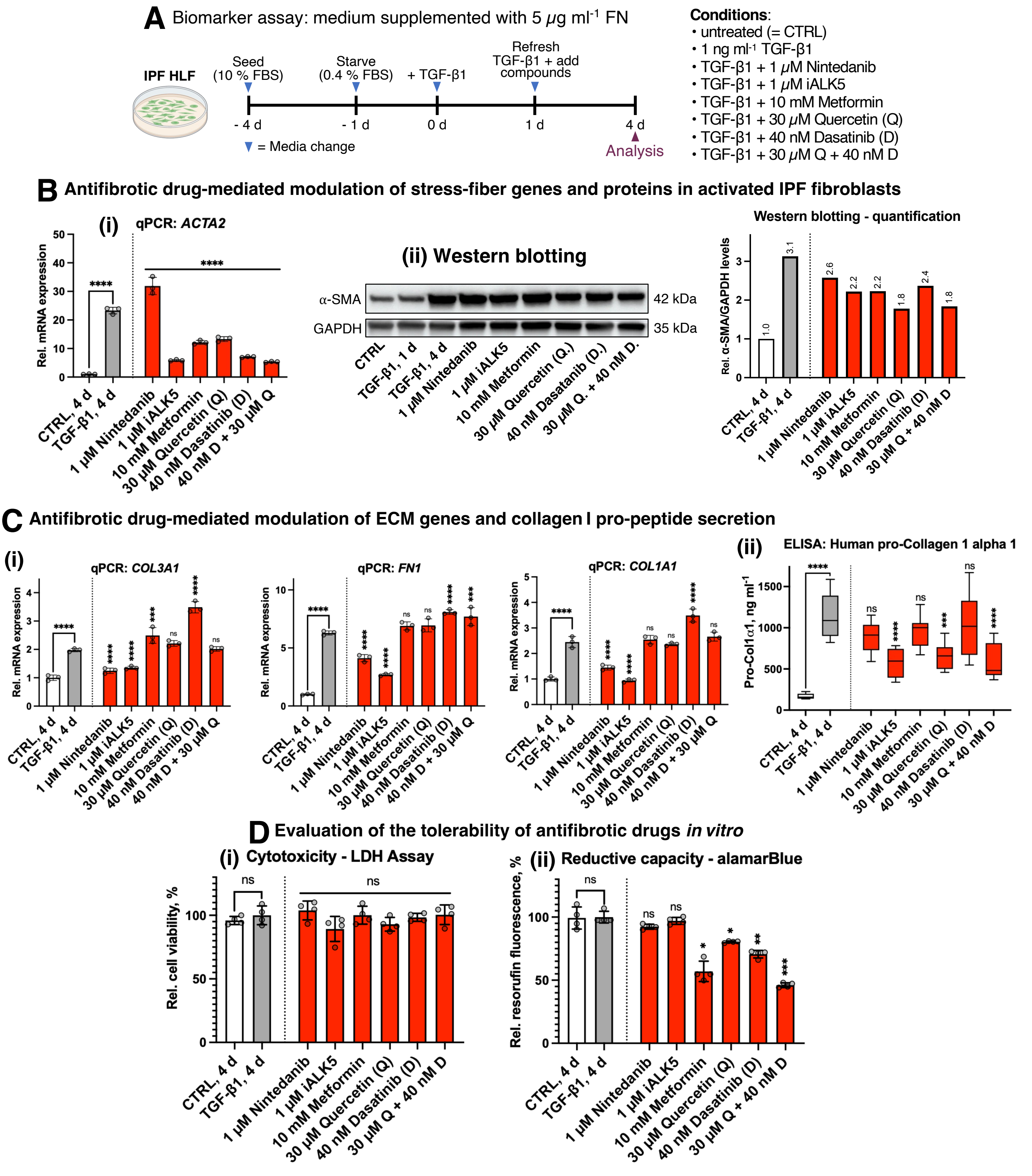


**Figure S10 | Effects of antifibrotic drugs on fibrotic markers in 2D-cultured, TGF-β1-activated IPF fibroblasts.**

**(A) Experimental setup.** IPF fibroblasts were seeded 3 days before serum starvation (day −1) and subsequently pre-activated with 1 ng ml$^{-1}$ TGF-$\beta_1$ for 1 day. Upon medium refresh, cells were treated for 3 days with the indicated compounds (± 1 ng ml$^{-1}$ TGF-β1): 1 μM nintedanib, 1 μM iALK5, 10 mM metformin, 30 μM quercetin (Q), 40 nM dasatinib (D), or 30 μM Q + 40 nM D.

**(B) Stress-fiber markers.** (i, mRNA) *ACTA2* expression quantified by RT-qPCR (technical triplicates); trends represent two independent experimental rounds. (ii, protein) α-SMA protein abundance determined by Western Blot and quantified as α-SMA/GAPDH ratios by densitometry; one representative blot of three independent rounds is shown.

**(C) ECM markers.** (i, mRNA) *COL3A1*, *FN1*, and *COL1A1* expression quantified by RT-qPCR (technical triplicates); trends represent two independent rounds. (ii, protein surrogate) Collagen I α1 N-terminal pro-peptide quantified by ELISA from culture supernatants. Data represent two independent rounds measured in two wells each with technical duplicates (total n = 8).

**(D) Cell viability.** Viability under drug treatment assessed by CyQUANT™ LDH-release assay (i) and alamarBlue assay (ii). Data represent two independent experimental rounds.

Across all repeated rounds, the same biological cell donor was used.

**Statistics.** Normality was assessed with the Shapiro–Wilk test and variance homogeneity with Levene's test. For normally distributed data with equal variances, significance was tested using unpaired, two-sided multiple-comparison t-tests with Benjamini–Hochberg correction (applied to B(i), C(i), and C(ii)). Due to non-normal distribution, D was analyzed using pairwise Wilcoxon rank-sum tests with Benjamini–Hochberg multiple-comparison correction. $P < 0.05$ was considered significant (* $p < 0.05$; ** $p < 0.01$; *** $p < 0.001$; **** $p < 0.0001$).

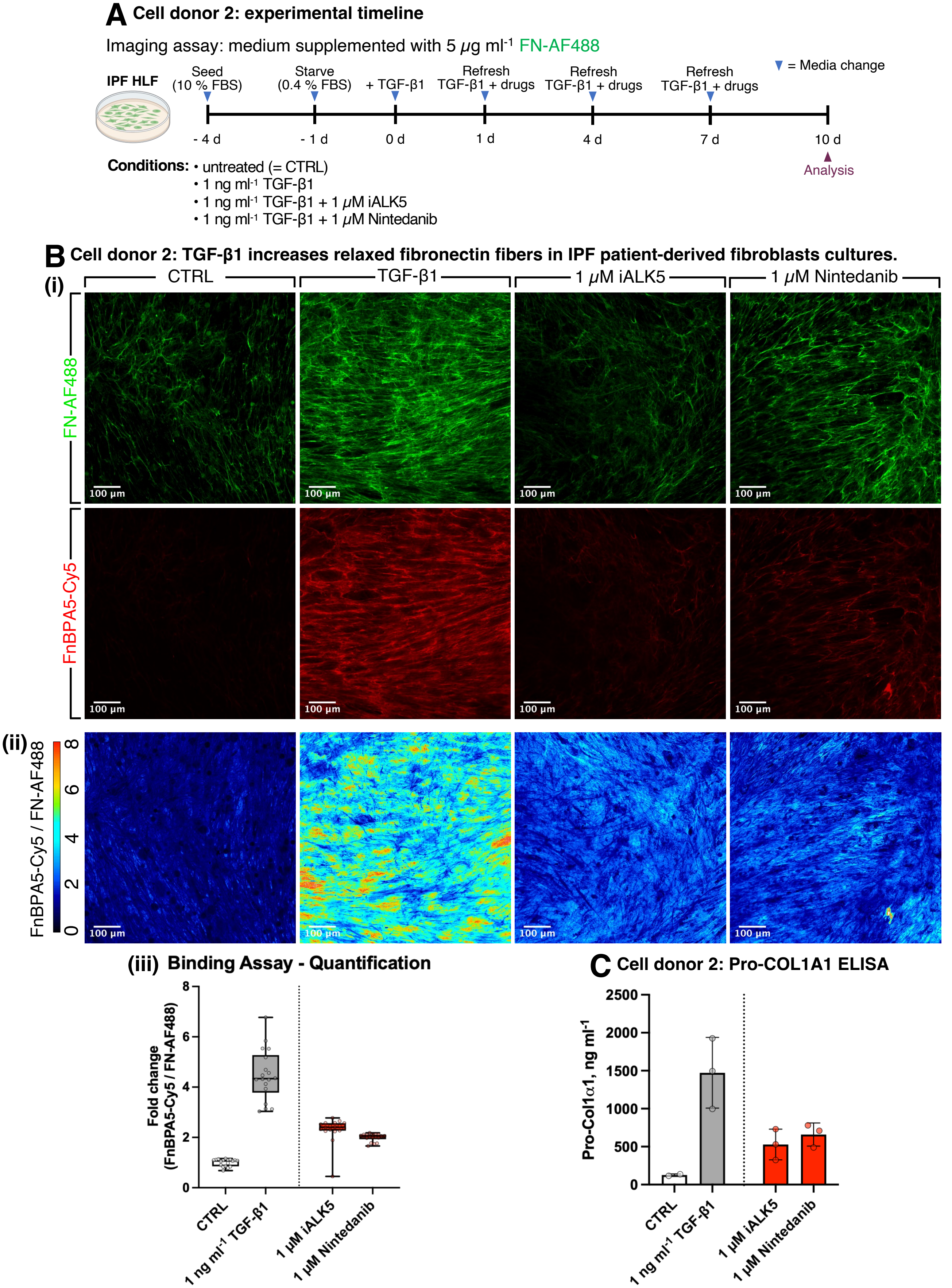


**Figure S11 I IPF cell donor 2 also forms fibrotic *in vitro* niches enriched in relaxed fibronectin fibers upon TGF-β1 stimulation.**

(A) Experimental timeline for donor 2 cells using three rounds of TGF-β1 + drug treatment (one initial dose, two refreshes). Conditions: untreated control (CTRL), TGF-β1 (1 ng ml$^{-1}$), and TGF-β1 + iALK5 (1 μM) or TGF-β1 + Nintedanib (1 μM).

(B) Binding assay confirming that TGF-β1 increases relaxed fibronectin fibers in IPF-derived fibroblasts from donor 2, consistent with the trend observed for donor 1 (Figure 3). (i) Representative single-channel confocal images of FN-AF488 and FnBPA5-Cy5 acquired with a 20× air objective on a Nipkow spinning-disk confocal microscope. (ii) Pixel-resolved ratiometric heatmaps derived from images in (i). (iii) Box plots showing all ROIs (6 wells per condition, 3 ROIs per well). Ratios represent FnBPA5-Cy5 / FN-AF488 averaged per ROI. Box plots: center line = median; box = 25th–75th percentile; whiskers = min–max.

(C) Collagen I pro-peptide α1 ELISA. Concentrations of the N-terminal collagen I pro-peptide α1 were measured from supernatants of independently seeded wells: 3 wells each for TGF-β1, TGF-β1 + iALK5, and TGF-β1 + Nintedanib; 2 wells for CTRL.

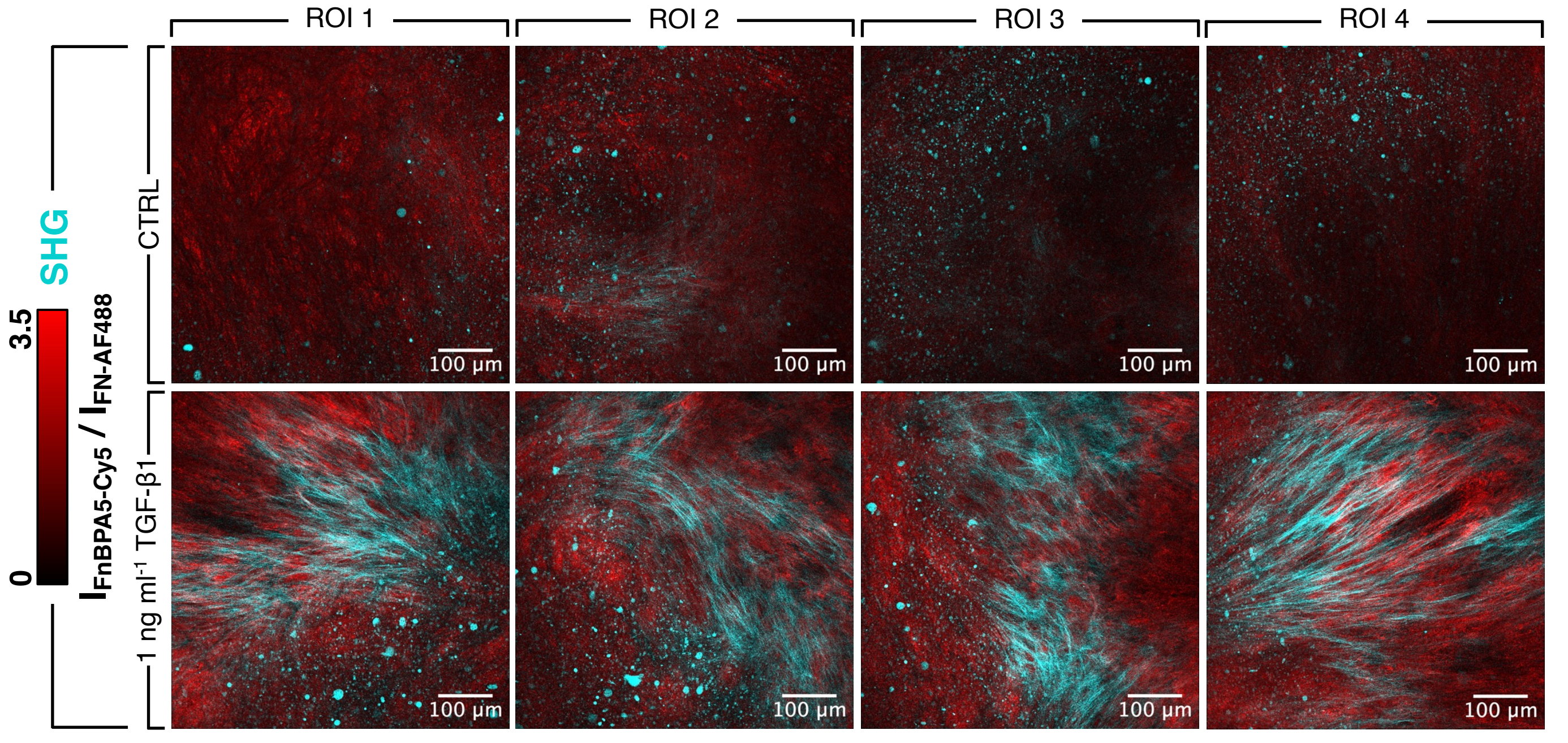


**Figure S12 | Co-localization of areas enriched in relaxed FN fibers and collagen fibrils in IPF fibroblasts from donor 2.**

IPF cells (donor 2) were cultured with FN-AF488 and treated without (CTRL, A) or with 1 ng $ml^{-1}$ TGF-β1 (B) (assay timeline as in Figure 3A). FnBPA5-Cy5, FN-AF488, and SHG signals were acquired using a Leica SP8 line-scanning confocal microscope (25×, 0.95 NA HCX IRAPO L water-immersion objective) in single-photon confocal mode for fluorophores and two-photon mode for SHG. While the correlation between relaxed fibronectin fibers (ratio) and SHG was established for donor 1 in Figure 3Bii using different xy positions within the same wells, these panels for donor 2 provide a direct spatial comparison at identical xy positions. To visualize this correlation, images are presented as cyan–red overlays: collagen fibrils (SHG signal) are shown in cyan, while the ratiometric FnBPA5-Cy5 / FN-AF488 signal (relaxed FN fibers) is shown in red (scale: 0–3.5; replacing the rainbow LUT use in Figure 3Bii for visual contrast). To generate these overlays despite longitudinal chromatic aberration inherent to two-photon microscopy, specific z-axis processing was applied: ratiometric heatmaps represent an average of five 1-$\mu$m-spaced z-planes (similar as Figure 3Bii), while the SHG signal is displayed for the plane of maximum intensity, which was determined and acquired after the fluorophore stack. This approach confirms that the relaxed FN fiber content increases together with SHG-positive collagen fiber content in the same regions. Four ROIs were imaged per condition and are presented.
